# Postnatal expansion of mesenteric lymph node stromal cells towards reticular and CD34^+^ stromal cell subsets

**DOI:** 10.1038/s41467-022-34868-4

**Published:** 2022-11-24

**Authors:** Joern Pezoldt, Carolin Wiechers, Mangge Zou, Maria Litovchenko, Marjan Biocanin, Michael Beckstette, Katarzyna Sitnik, Martina Palatella, Guido van Mierlo, Wanze Chen, Vincent Gardeux, Stefan Floess, Maria Ebel, Julie Russeil, Panagiota Arampatzi, Ehsan Vafardanejad, Antoine-Emmanuel Saliba, Bart Deplancke, Jochen Huehn

**Affiliations:** 1 Department Experimental Immunology, Helmholtz Centre for Infection Research, 38124 Braunschweig, Germany; 2 Laboratory of Systems Biology and Genetics, École Polytechnique Fédérale de Lausanne, 1015 Lausanne, Switzerland; 3 Department of Computational Biology for Individualised Medicine, Centre for Individualised Infection Medicine, Helmholtz Centre for Infection Research and Hannover Medical School, 30625 Hannover, Germany; 4 Genome Informatics Group, Bielefeld Institute for Bioinformatics Infrastructure, Department of Technology, Bielefeld University, 33615 Bielefeld, Germany; 5 Institute of Animal Breeding and Genetics, University of Veterinary Medicine Vienna, Vienna, Austria; 6 Core Unit Systems Medicine, University of Wuerzburg, 97080 Wuerzburg, Germany; 7 Helmholtz Institute for RNA-based Infection Research (HIRI), Helmholtz Center for Infection Research (HZI), 97080 Würzburg, Germany; 8 Cluster of Excellence RESIST (EXC 2155), Hannover Medical School, 30625 Hannover, Germany

**Keywords:** Epigenetics in immune cells, Differentiation, Lymph node, Transcriptomics

## Abstract

Gut-draining mesenteric lymph nodes (LN) provide the framework to shape intestinal adaptive immune responses. Based on the transcriptional signatures established by our previous work, the composition and immunomodulatory function of LN stromal cells (SC) vary according to location. Here, we describe the single-cell composition and development of the SC compartment within mesenteric LNs derived from postnatal to aged mice. We identify CD34^+^ SC and fibroblastic reticular stromal cell (FRC) progenitors as putative progenitors, both supplying the typical rapid postnatal mesenteric LN expansion. We further establish the location-specific chromatin accessibility and DNA methylation landscape of non-endothelial SCs and identify a microbiota-independent core epigenomic signature, showing characteristic differences between SCs from mesenteric and skin-draining peripheral LNs. The epigenomic landscape of SCs points to dynamic expression of Irf3 along the differentiation trajectories of FRCs. Accordingly, a mesenchymal stem cell line acquires a Cxcl9^+^ FRC molecular phenotype upon lentiviral overexpression of Irf3, and the relevance of Irf3 for SC biology is further underscored by the diminished proportion of Ccl19^+^ and Cxcl9^+^ FRCs in LNs of Irf3^-/-^ mice. Together, our data constitute a comprehensive transcriptional and epigenomic map of mesenteric LNSC development in early life and dissect location-specific, microbiota-independent properties of non-endothelial SCs.

## Abstract (web summary)

Lymph nodes in various locations of the body differ in their cell composition and gene expression signatures. Here authors show that the rapid postnatal expansion of lymph nodes is governed by CD34 ^+^ stromal cells and fibroblastic reticular stromal cell progenitors, distinguished by intrinsic, microbiome-independent core epigenetic blueprints.

## Introduction

The mammalian immune system is tasked to detect pathogenic incursions and maintain balanced immune responses. Failure to achieve equilibrium can result in the development of local and systemic overreactions to self and foreign antigens or higher susceptibility to infections. As lymph nodes (LN) are the initial hub translating early innate responses into lasting adaptive antigen-specific immunity, their tissue-specific modulation of developing immune responses is of essence to calibrate immune responses throughout life. LNs start to develop prenatally as early as embryonic day (E)13 in mice, in a process tightly regulated by mesenchymal lymphoid tissue organizer (LTo) and hematopoietic lymphoid tissue inducer (LTi) cells, yielding primordial LN anlagen^1,2^. Initial recruitment and retention of LTis are mediated by endothelial LTos^3^. The same endothelial LTos subsequently activate Cxcl13^+^ mesenchymal LTos, further recruiting additional LTis in a Cxcl13^+^-driven manner^2–4^. With the LN anlagen established by E17, containing a dense network of LTis and LTos^5^, endothelial and mesenchymal cells further proliferate. Already at E18, lymphatic endothelial cells have sufficiently expanded to envelope the core parenchyma of the LN, establishing the border to the surrounding tissue beneath the forming LN capsule^6^. After birth, the influx of T and B cells drastically increases, requiring and driving the rapid expansion of the LN stromal cell (LNSC) compartment including non-endothelial SCs.

The LN parenchyma becomes increasingly segregated as the B and T cell zones are establishing^7^. While the cortex situated underneath the subcapsular sinus of the LN contains the B cell follicles, T cells and dendritic cells interact in the paracortex beneath^8^. Aside blood and lymphatic endothelial cells, a third major SC population, the fibroblastic reticular stromal cells (FRC) populate the adult LN. The FRC pool consist of several heterogeneous subsets, including Cxcl13^+^ follicular dendritic cells, Cxcl13^+^ marginal reticular cells below the subcapsular sinus, and Ccl19^+^ T cell zone reticular cells and medullary FRCs located in the paracortex and medulla, respectively^4^. These FRCs are thought to develop from mesenchymal LTos transitioning through a myofibroblastic precursor stage^9^. Recent single-cell (sc)RNA-seq profiling of LNSCs revealed that the podoplanin (*Pdpn*)*-*expressing SC compartment encompasses a distinct and heterogeneous population of non-endothelial CD34^+^ SCs, which are located at the LN capsule or the adventitia of large vessels^10–12^. While several lineage-tracing models have delineated the prenatal origin of non-endothelial LNSCs^13–15^, the postnatal differentiation process of FRCs and CD34^+^ SCs along with the acquisition of immunomodulatory functions, presumably tightly regulated by epigenomic modifications and gene regulatory networks, is less well defined.

Although the initial priming of the adaptive immune response critically relies on antigen-presenting dendritic cells, the intrinsic microenvironment of the respective tissue-draining LN and its SC compartment greatly influences this process as well. Particularly, the heterogeneous population of FRCs has been shown to shape the adaptive immune responses by providing survival molecules such as IL-7 and BAFF^16,17^, or readily upregulating iNOS upon IFNγ signaling, thereby limiting the expansion of pro-inflammatory T cells and globally suppressing aberrant priming of adaptive immune cell differentiation^18,19^. In this context, the distinct localization of FRC subsets, such as Cxcl13^+^ follicular dendritic cells infrastructurally organizing B cell follicles and Ccl19^+^ T cell zone reticular cells facilitating dendritic cell and T cell interactions, is an important factor of the FRC-mediated immune modulation^4^. Another example are Cxcl9^+^ FRCs which mitigate effective migration of Cxcr3^+^ cells, including memory CD8^+^ T cells, during the course of anti-viral immune responses^12,20^. In addition to these immunomodulatory functions inherent to any LN, the local SC compartment also tissue-specifically shapes the migratory and effector properties of T cells^11,21,22^.

Despite the detailed understanding of prenatal LN development, little is known about which transcriptional regulators impinge postnatally on the presumably common pool of mesenchymal LTos to give rise to the heterogeneous population of non-endothelial SCs^23^ and their immune-modulatory potential^11,22,24^. Although multiple mechanisms through which mesenchymal SCs modulate the LN microenvironment have been identified, the underlying transcriptional regulators defining distinct functional SC responses from birth and throughout adulthood are only incompletely understood^25,26^.

Here, using scRNA-seq we describe the development of the SC compartment within mesenteric LNs (mLN) derived from postnatal to aged mice and observe a bifurcational postnatal segregation of potential mesenchymal progenitors. We map the location-specific, microbiota-independent transcriptional and epigenomic landscape of non-endothelial SCs, pointing to the dynamic expression of transcription factors (TF) in distinct SC subsets during ontogeny. Overall, our data provide a valuable resource to delineate transcriptional regulators that govern mesenteric LNSC development in early life.

## Results

### Developmental age constrains cellular and functional composition of SCs within mLNs

**Fig. 1:**
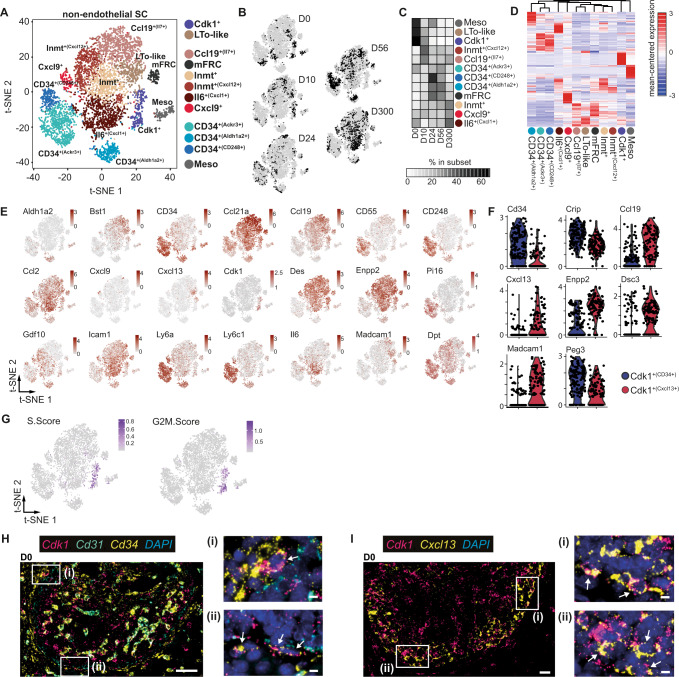
Postnatal ontogeny of non-endothelial SCs within mLNs. CD45^-^CD24^-^ cells were isolated from mLNs of day 0, 10, 24, 56, and 300 old SPF-housed mice and subjected to scRNA-seq. One replicate was performed for day 0, 10, 24, and 300 time points, and two replicates were generated for day 56 time point. Non-endothelial SCs were identified as non-LECs, non-BECs, and non-PvCs. **A** t-SNE plot of merged SCs across ages showing cluster segregation. **B** t-SNE plot of SCs from each age. **C** The heatmap represents the percentage of cells in each subset across time points normalized to cell number. **D** Hierarchical clustering of subsets based on the mean expression of the Top 40 DEGs per subset. **E** t-SNE plot colored for expression of segregating genes. **F** Violin plot of DEGs for subsets identified among putative postnatal progenitors. **G** t-SNE summarizing the expression of S-phase (left) and G2M-phase (right) associated genes as the cumulative Z-score. **H**, **I** Sections (3 µm) of D0 neonatal mLN were stained with the indicated RNAscope probes and imaged by fluorescence microscopy. Nuclei were counter-stained with DAPI (blue). Images were subjected to linear contrast enhancement. Images represent an overview of mLN anlagen with “white squares” indicating regions of interest. (i–ii) Images represent zoom-ins of respective regions of interest. Arrows indicate marker co-expression per cell. Representative tissue sections (*n* = 2–3). **H** Capsular positioning of *Cd34*^*+*^*Cdk1*^*+*^ cells. Overview (scale bar = 50 µm) and zoom-in (scale bar = 5 µm) for RNA probes specific to *Cdk1, Cd31*, and *Cd34*. **I** Cortex-localized *Cxcl13*^*+*^*Cdk1*^*+*^ cells. Overview (scale bar = 20 µm) and zoom-in (scale bars = 5 µm) for RNA-probes specific to *Cdk1* and *Cxcl13*. BEC blood endothelial cell, LEC lymphatic endothelial cell, LTo-like lymphoid tissue organizer like cell, Meso mesothelial-like cells, mFRC metabolically active fibroblastic reticular stromal cell, PvC perivascular cell, SC stromal cell.

Recent evidence suggests that mLNs undergo rapid postnatal expansion and acquire their unique functional properties already in early life^25,26^. Thus, we here first aimed to dissect changes in SC composition along mLN development during this critical period. To this end, we resected mLNs from mice at early postnatal age (day 0/1, D0), early (D10), and late (D24) juvenile stages, adult age (D56), as well as old age (D300), and obtained FACS-purified CD45^-^CD24^-^ cells. Subsequently, we performed single-cell (sc)RNA sequencing and gathered transcriptomes of 15,659 cells with comparable sequencing depth (55,000–98,000 mean reads per cell) across the different time points. Initially, we used transcriptional signatures for mLNSCs^11^ and the canonical marker for endothelial cells *Pecam1* (Supplementary Fig. 1A) to remove endothelial cells and perivascular cells from the analysis after alignment of samples using diagonal canonical correlation analysis (see “Methods”). In addition, we observed and excluded cells that were disproportionately represented in D0/1 mLNs, expressing significantly higher levels of the growth factors *Igf1* and *Igf2* as well as the extracellular matrix protein *Mfap4*, indicative of adjacent tissue fibroblasts (Supplementary Fig. 1B, C, Supplementary Data 1). To obtain an unbiased picture, we re-embedded 5658 non-endothelial SCs across all five developmental stages. Twelve transcriptional clusters harboring unique functional properties were identified based on differentially expressed genes (DEG), gene ontology (GO) analysis, and previously published signatures^11,12^ (Fig. 1A, Supplementary Fig. 1D, E, Supplementary Data 2). These clusters were broadly separated into CD34^+^ SCs, including CD34^+(CD248+)^, CD34^+(Ackr3+)^, CD34^+(Aldh1a2+)^, metabolically active FRC, Il6^+(Cxcl1+)^ FRC, Cxcl9^+^ FRC, Inmt^+^ FRC, Inmt^+(Cxcl12+)^ FRC and Ccl19^+(Il7+)^ FRC, mesothelial-like, LTo-like and Cdk1^+^ subsets (Fig. 1A). All identified clusters were variably represented along postnatal mLN development, with particularly LTo-like and Cdk1^+^ subsets being over-represented at D0 and D10 (Fig. 1B, C). As expected, LTo-like SCs highly expressed *Cxcl13*, *Tnfsf11* and *Madcam1* (Fig. 1D, E)^2,3,9^. Interestingly, the Cdk1^+^ subset could be further subdivided into two distinct populations (Fig. 1F). Cdk1^+(Cxcl13+)^ cells expressed significantly higher levels of canonical markers for LTo-like cells including *Madcam1* and *Ccl19*, indicative of a close relation to previously described LTos and their propensity to function as progenitors during LN development^27^. Cdk1^+(CD34+)^ cells showed higher expression for several collagens (*Col3a1, Col6a2,* and *Col1a2*) as well as genes involved in regulating Bone Morphogenetic Protein signaling, including *Gpc3* and *Crip* (Fig. 1F)^28^. Both Cdk1^+(Cxcl13+)^ and Cdk1^+(CD34+)^ cells are highly proliferative, not only because of the expression of the namegiving *cyclin-dependent kinase 1* (*Cdk1*), a key kinase at the transition to the S-phase (Fig. 1E), but also reflected in their high S.Score and G2M.Score when compared to all other subsets (Fig. 1G). Yet, the highly proliferative state of these cells is not causative for their clustering since the S.Score and G2M.Score were among the covariates regressed out during the analysis of the scRNA-seq data (see “Methods”).

Based on the findings that both Cdk1^+^ subsets were over-represented at early stages (D0 and D10, Fig. 1B, C) and display a highly proliferative state, we hypothesized that these cells might represent progenitor-like cells present in distinct niches of the developing LN anlagen around birth. We aimed to identify the localization of the respective putative progenitor populations within the developing LNs by utilizing RNAscope on neonatal mLN tissue slices. Initially, we confirmed that the RNA integrity of D0 mLN remained intact after resection by staining for a mix of ubiquitously expressed genes (*Polr2a, Ubc, Ppib*) as a positive control (Supplementary Fig. 2A) and that the majority of *Ccl19* and *Cxcl13* expressing cells were spatially separated from *Cd34*^*+*^ cells at this stage of postnatal mLN development (Supplementary Fig. 2B, C). Next, we utilized *Cdk1* expression to localize progenitor-like Cdk1^+^ cells. We readily identified capsular *Cd34* and *Cdk1* expressing non-endothelial *Cd31*^-^ cells (Fig. 1H). Importantly, *Cd34*^+^*Cdk1*^+^ cells were neither present within the cortical nor paracortical areas, but could be identified within adjacent tissue (Supplementary Fig. 2D). As expected^3,4^, we furtheron detected *Cdk1*^*+*^*Cxcl13*^*+*^ cells predominantly in the developing cortex (Fig. 1I).

Together, these data delineate the postnatal expansion of the heterogeneous non-endothelial SC compartment and identify two putative postnatal progenitor cell populations that are distinctly positioned within the developing LN.

### Distinct progenitors establish the non-endothelial SC compartment rapidly after birth

**Fig. 2:**
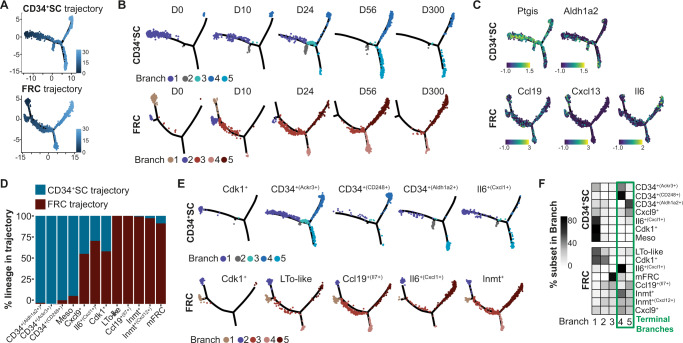
Distinct postnatal progenitors give rise to FRCs and CD34^+^ SCs. CD45^-^CD24^-^ cells were isolated from mLNs of day 0, 10, 24, 56, and 300 old SPF-housed mice and subjected to scRNA-seq. One replicate was performed for day 0, 10, 24, and 300 time points, and two replicates were generated for day 56 time point. Non-endothelial SCs were identified as non-LECs, non-BECs, and non-PvCs. **A** Pseudotime ordering of FRCs and CD34^+^ SCs. **B** Pseudotime trajectories superimposed with cells per time point. **C** Gene expression on pseudotime trajectories. **D** Bar graph depicts the proportion of cells from the indicated subset within the FRC or CD34^+^ SC trajectory. **E** Cells per cell subset across branches superimposed on pseudotime trajectories. **F** The heatmap represents the percentage of cell subsets across branches normalized to cell number. BEC blood endothelial cell, FRC fibroblastic reticular stromal cell, LEC lymphatic endothelial cell, mLN mesenteric lymph node, PvC perivascular cell, SC stromal cell, SPF specific pathogen-free.

Previous studies showed that postnatal expansion of non-endothelial SCs within the LN relies on the progenitor potential of LTo cells, differentiating into various SC subsets^14,29,30^. In addition, progenitors with LNSC potential have been shown to reside in the CD34^+^ perivascular niche of multiple adult organs^10^. Thus, we were keen to investigate how the Cdk1^+(Cxcl13+)^ and Cdk1^+(CD34+)^ cells fit into the postnatal developmental trajectory and to which specific SC subsets they would give rise to. We utilized trajectory analysis to further elucidate the precursor potential of the Cdk1^+(Cxcl13+)^ and Cdk1^+(CD34+)^ cells observed at early postnatal mLN development and to identify putative precursor-progeny relationships. We initially constructed a trajectory using all non-endothelial SCs, and observed two distinct sets of branches evolving along physiological development (Supplementary Fig. 3A), underscored by the expression of key marker genes identifying progenitors at distinct, presumably early stages of development (Supplementary Fig. 3B)^27,31,32^, indicating a bifurcational development. We therefore re-embedded cells situated along the CD34^+^ SC or FRC trajectory (Supplementary Fig. 3C) and employed pseudotime mapping using Monocle2^33^. As expected, early developmental time points corresponded to pseudotemporal branches at the starting point of the trajectory (Fig. 2A, B). Importantly, already at D24, CD34^+^ SCs were distributed along the complete pseudotemproal space, whereas only minor changes in the distribution of cells across pseudotime were observed beyond D56, indicating that main non-endothelial SC differentiation is established shortly after weaning (Fig. 2A, B). While *Ccl19* and *Ptgis* were constitutively expressed over the developmental trajectory, subset-defining genes including *Aldh1a2* and *Il6* were predominantly expressed at distinct branches of the CD34^+^ SC or FRC trajectories, respectively (Fig. 2B, C), indicative of developmental segregation for each subset of CD34^+^ SCs and FRCs, culminating in distinct functional subsets (Fig. 2D). Importantly, *Cxcl13* was expressed highest early during development in FRCs (Fig. 2C). As expected, pseudotime starting points of both the CD34^+^ SC and FRC trajectories comprised predominantly Cdk1^+(CD34+)^ or Cdk1^+(Cxcl13+)^ cells (Fig. 2E). Within the FRC trajectory, Cdk1^+(Cxcl13+)^ cells pseudotemporally aligned with LTo-like cells, indicating that these cells are of similar origin, giving rise to two major branches of differentiation that correspond to different subsets of FRCs, namely Ccl19^+(Il7+)^ and Il6^+(Cxcl1+)^ (Fig. 2E, F). Distinct differentiation paths were underscored by an increased expression of *Tnfsf13b, Il33, Ccl19*, and *Tgfbi* for Ccl19^+(Il7+)^, and *Cxcl1* and *Il6* for Il6^+(Cxcl1+)^ subsets along pseudotemporal development (Fig. 2C, Supplementary Fig. 3D).

In line with the FRC trajectory, the CD34^+^ SC development initiated from Cdk1^+(CD34+)^ cells branched into two major axes consisting predominantly of either CD34^+(CD248+)^ or CD34^+(Aldh1a2+)^ cells (Fig. 2E, F). As expected, a small proportion of CD34^+^ SCs^2,10^, was already in place at D0, rapidly expanding at D10 and D24 (Fig. 2B). As anticipated, based on the heterogenous composition of the mLN’s non-endothelial SC compartment, the main terminal branches showed distinct expression profiles. While the CD34^+(CD248+)^ branch expressed higher levels of *Ptgs1* and *Ptgis* enabling prostacyclin synthesis (Fig. 2C, Supplementary Fig. 3D), the CD34^+(Aldh1a2+)^ branch expressed higher levels of *Col15a1* and *Vtn* together with *Fgf7* and *Gdf10* potentially generating a specific cell adhesion environment (Supplementary Fig. 3D). Surprisingly, cells of the Cxcl9^+^ subset were distributed similarly between FRCs and CD34^+^ SCs and were not over-represented at a specific terminal branch, neither in the FRC nor the CD34^+^ SC trajectory (Fig. 2D). The dispersion of the Cxcl9^+^ subset across the two main subsets and the trajectory is underscored by the unique expression of *Ly6a*, also a core feature of CD34^+^ SCs, but the lack of consistent *Cd34* expression (Fig. 2E). In addition, as the majority of SC expansion appears to take place before weaning, we decided to focus the analysis on the early time points and only embed non-endothelial SCs isolated at D0, D10, and D24 to conduct pseudotime mapping with Monocle2 on this cell pool. Again, we observed two sets of branches evolving from D0 to D10 (Supplementary Fig. 4A, B). Both sets of branches developed from Cdk1^+^ cells, with one set of branches containing mainly FRC cluster cells and the other set containing CD34^+^ SC subsets (Supplementary Fig. 4C, D). Cxcl9^+^ cluster cells showed no clear polarization and were distributed across the whole trajectory (Supplementary Fig. 4D). Upon re-embedding the distinct branch sets into an FRC or CD34^+^ cell trajectory, Cdk1^+^ cluster cells once again formed the starting points for each of the trajectories (Supplementary Fig. 4E, F). Thus, the trajectories based on non-endothelial SCs isolated before weaning closely resemble the trajectory obtained when cells from all time points were embedded and pseudotemporally ordered, underscoring the putative precursor-progeny relationships.

Viewed as a whole, the pseudotemporal dissection of the non-endothelial SC compartment indicates that postnatal expansion of the main SC populations within mLNs is likely driven by two proliferating progenitor populations, both of which are already postnatally set on a defined FRC or CD34^+^ SC differentiation trajectory (Supplementary Fig. 4G).

### Microbial colonization does not shape the non-endothelial SC subset composition

**Fig. 3:**
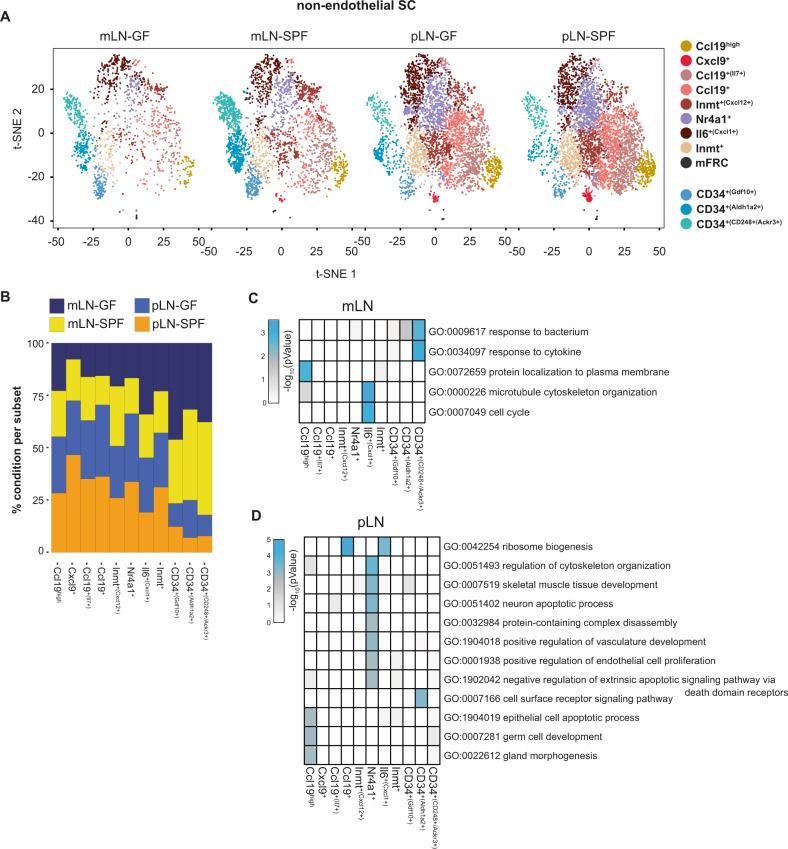
Non-endothelial SC subset composition is largely unaffected by microbial colonization. CD45^-^CD24^-^ cells were isolated from mLNs and pLNs of 6–10 weeks old GF or 7–12 weeks old SPF-housed mice and subjected to scRNA-seq. The scRNA-seq dataset for SPF mice (two replicates per condition) was previously published^11^. One replicate for pLN and mLN from GF mice was newly processed. Non-endothelial SCs were identified as non-LECs, non-BECs, and non-PvCs. **A** t-SNE plot depicting cluster segregation of non-endothelial SC subsets between GF and SPF mice for mLN (left) and pLN (right). **B** Bar graph showing the proportion of cells within each subset normalized to total cell number belonging to either mLN-GF, mLN-SPF, pLN-GF, or pLN-SPF condition. **C** GO analysis of biological processes of upregulated DEGs identified between SPF-mLN and GF-mLN condition (enrichment analysis of GO terms calculated with Fisher´s exact test). **D** GO analysis of biological processes of DEGs identified between SPF-pLN and GF-pLN condition (enrichment analysis of GO terms calculated with Fisher’s exact test). BEC blood endothelial cell, DEG differentially expressed gene, FRC fibroblastic reticular stromal cell, GF germ-free, GO gene ontology, LEC lymphatic endothelial cell, mLN mesenteric lymph node, pLN peripheral skin-draining lymph node, PvC perivascular cell, SC stromal cell, SPF specific pathogen-free.

Aside from changes in non-endothelial SC subset composition during mLN development, we were also keen to ascertain compositional changes caused by external mitigators such as the microbiota. Due to the close proximity of mLNs to the gut, we hypothesized that SCs within mLNs might be uniquely shaped by the microbiota in comparison to SCs from peripheral skin-draining LNs (pLN). Indeed, we could already previously demonstrate that the SC subset composition differs between pLN and mLN, and identified minor transcriptional changes between SCs from germ-free (GF) and specific pathogen-free (SPF) mice on the bulk RNAseq level, pointing towards potential subset-specific changes wrought by microbial mediators^11^. To address this hypothesis, FACS-purified CD45^-^CD24^-^ cells from mLNs or pLNs of adult GF mice were subjected to scRNA-seq analysis, after which we performed a combined analysis of 18,045 cells from mLN and pLN of SPF and GF mice^11^. After removing endothelial cells and perivascular cells, 14,307 non-endothelial SCs were re-embedded. We identified twelve transcriptional clusters based on DEGs and previously published signatures^11,12^, namely Ccl19^high^ FRC, Cxcl9^+^ FRC, Ccl19^+(Il7+)^ FRC, Ccl19^+^ FRC, Inmt^+(Cxcl12+)^ FRC, Nr4a1^+^ FRC, Il6^+(Cxcl1+)^ FRC, Inmt^+^ FRC, metabolically active FRC, CD34^+(Gdf10+)^ SC, CD34^+(Aldh1a2+)^ SC, and CD34^+(CD248+/Ackr3+)^ SC. Surprisingly, the absence of microbiota did not overtly influence the non-endothelial SC subset composition neither in mLN nor in pLN analyzed as controls (Fig. 3A, B). While we observed mild tendencies for subset abundance between Cxcl9^+^ FRC, CD34^+(Gdf10+)^ SC, and CD34^+(Aldh1a2+)^ SC in mLN and for the latter also in pLN, all changes were only marginal (Fig. 3A, B). Next, we investigated the impact of microbiota on FRC and CD34^+^ SC subset functionality via GO analysis of the DEGs (log2FC: 0.2, padj: 0.05) identified on a per subset basis comparing GF and SPF condition per LN. For mLN, in total 129 upregulated DEGs were identified across all subsets and as anticipated, due to the absence of microbes in GF mice, the term GO:0009617 *response to bacterium* was enriched particularly in CD34^+(CD248+/Ackr3+)^ cells (Fig. 3C). Thus, the CD34^+(CD248+/Ackr3+)^ cluster is likely positioned to respond to microbiota as this subset is also more abundant in mLN compared to pLN. For pLN, 642 upregulated DEGs were identified, but none of the enriched GO terms could be associated to a response to microbiota (Fig. 3D).

In summary, while minor changes in composition of certain FRC and CD34^+^ SC subsets were detected and CD34^+(CD248+/Ackr3+)^ cluster cells likely respond to microbial cues in mLN, overall the non-endothelial compartment is not drastically transcriptionally shaped by the microbiome and all SC subsets are also present in LNs of GF mice.

### Location defines the epigenomic landscape of non-endothelial LNSCs

**Fig. 4:**
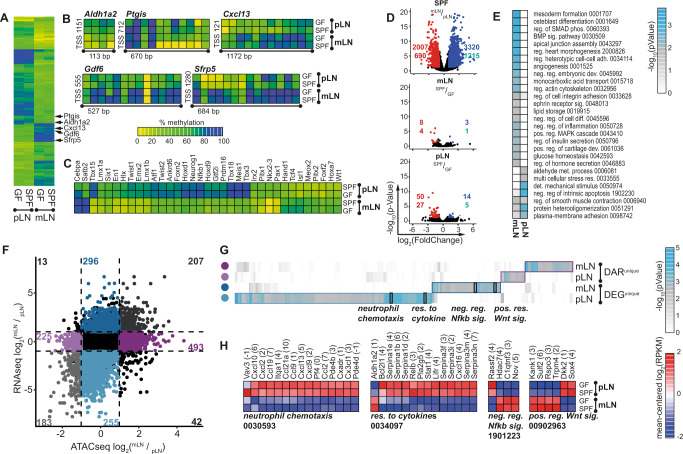
LN location defines the epigenomic landscape of non-endothelial SCs. CD45^-^CD24^-^CD31^-^Pdpn^+^ non-endothelial SCs were isolated from mLNs and pLNs of 6–10 weeks old GF or 7–12 weeks old SPF mice, and WGBS (**A**–**C**), ATAC-seq (**D**–**G**), or RNA-seq (**F**–**H**) analyses were performed. **A**–**C** DMRs were identified in colonization- (SPF vs. GF) and location-dependent (mLN vs. pLN) pairwise comparisons (2–3 replicates per condition). Scale bar depicts the extent of methylation with 100% being fully methylated and 0% being non-methylated. **A** The heatmap represents the mean methylation of significant DMRs within the promotor region of 286 genes. **B** Heatmaps represent CpG methylation of exemplary DMRs. The distance from the TSS is indicated and the size of genomic loci denoted in base-pairs (bp). **C** The heatmap represents the mean methylation of significant DMRs within the promotor region of TFs. **D**, **E** DARs were identified in colonization- (SPF vs. GF) and location-dependent (mLN vs. pLN) pairwise comparisons (3 replicates per condition). **D** Volcano plots of mean ATAC-seq FPKM comparing indicated samples. The number of DARs (top) and genes (bottom) is indicated per comparison. **E** GO analysis of biological processes of genes with location-dependent DARs (enrichment analysis of GO terms calculated with Fisher´s exact test). The numbers denote GO identifiers. **F**–**H** DEGs (3 replicates per condition) and DARs were identified in mLN vs. pLN pairwise comparisons. **F** Colored numbers in the scatterplot represent the number of genes with DAR and/or differential expression. Only genes with accessible loci within the promotor region were included in the analysis. On the x-axis, log_2_(FC) of accessibility per DAR is plotted and on the y-axis, the log_2_(FC) of gene expression for the comparison of mLN vs. pLN. **G** GO analysis of biological processes of DEGs and/or genes with at least one DAR (enrichment analysis of GO terms calculated with Fisher´s exact test). **H** Heatmaps represent the expression of all DEGs within the GO groups highlighted in (**G**). The numbers denote GO identifiers. ATAC-seq assay for transposase accessible chromatin sequencing, DAR differentially accessible region, DEG differentially expressed gene, det detection, DMR differentially methylated region, FC fold change, FPKM fragments per kilobase of peak per million reads, GF germ-free, GO gene ontology, mLN mesenteric lymph node, met metabolism, neg negative, pLN peripheral skin-draining lymph node, pos positive, reg regulation, SC stromal cell, sig signaling, SPF specific pathogen-free, TF transcription factor, TSS transcription start site, WGBS whole-genome bisulfite sequencing. Source data are provided in a Source data file.

Next, we aimed to study if the presence of microbiota modifies non-endothelial SCs at the epigenetic level. Moreover, as previous studies underlined the immunomodulatory functions of LNSCs^31^ we aimed to delineate epigenomic modifications that underlie the immunomodulatory function of LNSCs. To this end, we performed whole-genome bisulfite sequencing (WGBS) and assay for transposase accessible chromatin sequencing (ATAC-seq) to identify CpG methylation and genomic accessibility, respectively, of CD31^-^Pdpn^+^ non-endothelial SCs isolated by FACS from mLNs and pLNs originating from adult mice housed under SPF or GF conditions. We first performed DNA methylation analyses and we were able to determine the methylation status of 94.4% of the 2.19*10^7^ CpGs (Supplementary Fig. 5A). In total, we could identify 1532 non-overlapping differentially methylated regions (DMR) across all pairwise comparisons. Importantly the vast majority of the DMRs were location-dependent (Fig. 4A), whereas only 16 and 17 were commensal-dependent for pLN and mLN, respectively (Supplementary Data 3). Principal component analysis (PCA) and hierarchical clustering of individual samples based on DMRs with distinct genomic locations demonstrated that LN location predominantly influences the methylation status. While samples also mostly clustered according to SPF or GF origin, the segregation was rather poor (Supplementary Fig. 5). Over 390 DMRs, located in the proximity of the transcriptional start site, were annotated to 286 genes, including microenvironmental mediators (e.g., *Cxcl13, Gdf6, Sfrp5*)^34^, immunomodulatory enzymes (e.g., *Aldh1a2, Ptgis*)^35–37^, and TFs (e.g., *Isl1, Hoxd1, Meis1, Meis2, Nkx2-3, Tcf4, Foxn2*), potentially impinging on transcriptional regulation (Fig. 4B, C)^38^.

We then proceeded to obtain a global overview of chromatin accessibility using the obtained ATAC-seq data. Peaks were called per sample and merged to obtain common open genomic regions across all conditions comprising a total of 42,434 peaks (Supplementary Data 4–5), with the majority of the detected peaks being located at transcriptional start sites (Supplementary Fig. 6). Upon comparison to ENCODE database^39,40^ entries, chromatin accessibility profiles of non-endothelial SCs from LNs showed no close resemblance to any other tissue (Supplementary Fig. 7). As expected, we identified a substantial number of 5327 differentially accessible regions (DAR) between mLN-SPF and pLN-SPF, whereas absence or presence of microbiota only marginally influenced the accessibility profile of non-endothelial SCs for both mLN and pLN, corroborated by the low number of detected DARs (11 and 64, respectively; Fig. 4D). GO analysis of the location-dependent DARs revealed that SCs from mLNs are responsive to Bone Morphogenetic Protein signaling and negatively regulate inflammatory processes (Fig. 4E). Altogether, these data implicate that the tissue-specific location of LNs strongly influences the epigenomic landscape of non-endothelial SCs, thereby potentially contributing to TF-controlled gene expression programs.

We were then prompted to further dissect transcriptional and epigenomic co-regulation. In order to compare both transcriptome and chromatin accessibility or DNA methylation level, we obtained RNA-seq data of CD31^-^Pdpn^+^ non-endothelial SCs isolated from mLNs and pLNs originating from adult mice housed under SPF conditions. Surprisingly, there was only a minor overlap of genes associated with a demethylated DMR and elevated gene expression, with ten genes being upreglated in mLN and 20 genes being upregulated in pLN (Supplementary Fig. 8). We next correlated location-dependent differential expression with the respective chromatin accessibility within the promotor region on a per-gene basis, which allowed us to divide genes into three distinct groups (Fig. 4F). The first group includes genes whose transcriptional activity correlates with chromatin accessibility, with 207 and 183 for mLNSCs and pLNSCs, respectively. These genes comprise 24% of the DEGs and include *Nkx2-3*, *Ccl20*, and *Cxcl9*, known for their location-dependent differential expression^41^. *Nkx2-3*, which was previously reported to be required for lymphoid organ and intestinal development^42–44^, is substantially more accessible in mLN compared to pLN (Supplementary Fig. 6B). The second group contains 43% of the regulated genes that are differentially accessible, but not differentially expressed, potentially composed of elements that can respond to cellular activation. Therefore, we defined them as “inducible genes”. Interestingly, the third group, containing 296 genes for mLNSCs and 255 genes for pLNSCs, are differentially expressed but have no associated DARs, indicating that these genes are regulated by TFs that exploit already accessible chromatin. We here define them as “active TF regulated genes” (Fig. 4F). We performed GO analysis for biological processes on the “inducible genes” or “active TF regulated genes” for mLNSCs and pLNSCs. Within mLNSCs, GO terms indicative of immune suppression were enriched, including *negative regulation of Nfkb signaling* (Fig. 4G, H), while particularly for the genes associated with active TFs in pLNSCs, a substantial enrichment of GO terms associated with elevated immune responses, including *neutrophil chemotaxis* and *response to cytokine*, was identified (Fig. 4G, H). To validate part of these findings, flow cytometric analyses were performed and confirmed an increased frequency and significantly higher number of neutrophils in pLN when compared to mLN (Supplementary Fig. 9), mirroring the results from the GO term analysis. Although these findings do not formally prove a causal link between these cell types, they further suggest that non-endothelial SCs from pLN and mLN might differentially regulate neutrophil abundance under steady-state conditions. Yet, it is worth mentioning that neutrophils are generally very rare in LNs under homeostasis, and previously published studies had reported a similar frequency and number of neutrophils at different anatomic locations^45–47^.

Together, we could show that tissue location of the LNs substantially contributes to differences in DNA methylation and chromatin accessibility. In line with the scRNA-seq data, these location-dependent epigenomic differences overarche changes wrought by microbiota.

### TFs shape the subset-specific epigenomic landscape early during ontogeny

**Fig. 5:**
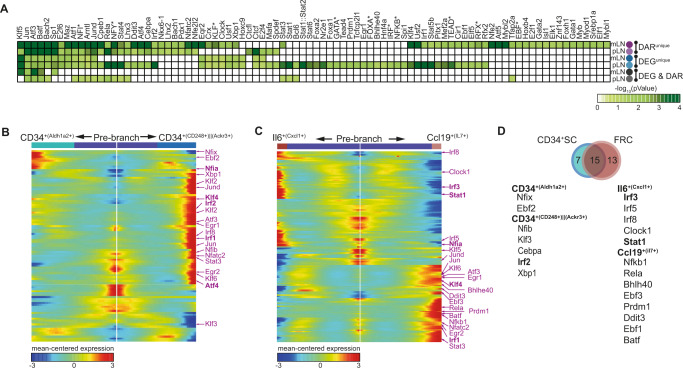
TFBS accessibility aligns with dynamically regulated TFs to shape differentiation and function of key SC subsets within mLNs. **A** CD45^-^CD24^-^CD31^-^Pdpn^+^ non-endothelial SCs were isolated from mLNs and pLNs of 7–12-week-old SPF mice. Subsequently, RNA-seq or ATAC-seq analyses were performed (see Fig. 4 for details). The enrichment of known TFBS motifs for each of the quadrants in Fig. 4F was utilized to identify TFBS over-represented in the accessible chromatin. The heatmap represents the *p*-value for enriched TFBS and its corresponding putative TF(s). Putatively binding TF families are indicated with an asterix. **B**, **C** CD45^-^CD24^-^ cells were isolated from mLNs of day 0, 10, 24, 56, and 300 old SPF-housed mice and subjected to scRNA-seq. Non-endothelial SCs were identified as non-LECs, non-BECs and non-PvCs (see Fig. 1 for details). Heatmap showing expression of detected TFs identified in (**A**) over pseudotime involved in the differentiation of **B** CD34^+^ SCs and **C** FRCs. TFs highlighted in “pink” are derived from TFBS enrichment analyses of LNSC DARs. **D** The Venn diagram depicts the overlap of accessibility-derived TFs. TFs upregulated per subset/branch are denoted in “black”. BEC blood endothelial cell, DAR differentially accessible region, det detection, DMR differentially methylated region, LEC lymphatic endothelial cell, mLN mesenteric lymph node, pLN peripheral skin-draining lymph node, FRC fibroblastic reticular stromal cell, PvC perivascular cell, SC stromal cell, TF transcription factor, TFBS transcription factor binding sites.

Next, we aimed to identify the TFs responsible for altering expression without modifying chromatin accessibility under steady-state conditions. To this end, we identified over-represented TF binding sites (TFBS) and their putative TFs, for the “inducible genes” and “active TF regulated genes” within accessible genomic loci of the respective genes. As expected, a substantial number of TFs, namely *Klf5, Jun, Atf3, Batf, Bach2*, *Sp1, Atf1, Nf1*, and *Jund* were enriched in at least five out of six gene loci sets, indicating their general involvement in shaping the function of non-endothelial SCs (Fig. 5A, Supplementary Fig. 10). However, there were also differences between mLN and pLN regarding the accessibility and enrichment of TFBS (Fig. 5A). We hypothesized that TFs identified from footprinting analyses of the accessible chromatin could also be dynamically expressed along mLN development. To this end, we combined the over-represented TFBS and their putative TFs within accessible genomic loci (Fig. 5A) with the trajectoral expression of all TFs detected in the developmental scRNA-seq analysis of CD34^+^ SCs and FRCs (Fig. 2). This allowed us to explore the TF binding motif enrichment in the epigenomic landscape of mLNSCs and investigate which TFs are expressed at key branching points of the FRC and CD34^+^ SC differentiation trajectories.

In total, 30% of the TFs identified from the accessible chromatin were differentially expressed over the developmental trajectory, of which 7 and 13 TFs were uniquely differentially expressed at the key branching point for CD34^+^ SCs and FRCs, respectively (Fig. 5B–D). TFs uniquely differentially expressed at the key branching point for CD34^+^ SCs were *Nfix, Ebf2, Nfib, Klf3, Cebpa, Irf2, Xbp1*, while TFs uniquely differentially expressed at the key branching point for FRCs were *Irf3/5/8, Clock1, Stat1, Nfkb1, Rela, Bhlh40, Ebf3, Prdm1, Ddit3, Ebf1*, and *Batf* (Fig. 5B–D). Of note, as only DEGs between branches are indicated, TFs identified in Fig. 5A may be expressed in general, but are not indicated. TFs falling into this category, which were identified for both CD34^+^ SCs and FRCs, include *Nfia, Egr1, Egr2, Stat3*, and *Atf4*. Particularly, the commonly over-represented TFs (Fig. 5A) are expressed at the key branching point of both CD34^+^ SC and FRCs (Fig. 5B, C), including *Atf3, Egr1, Egr2, Irf1, Jun, Jund*, and *Klf9*, indicating that these TFs shape the general course of chromatin accessibility of differentiating SCs (Fig. 5B–D, Supplementary Fig. 3E).

We next utilized *dynGENIE3*^48^ to identify key TFs that might be of functional relevance for the expanding LNSC subsets, and constructed a gene regulatory network of dynamically regulated genes encompassing TFs for each of the two terminal branches for both FRCs and CD34^+^ SCs. In line with the branch-point-based analysis, we observed that 23% of the dynamically regulated TFs were co-regulated consistently across FRCs and CD34^+^ SCs, including *Nfia*, *Egr2*, and *Ebf2* (Supplementary Fig. 11A, B). Despite the substantial proportion of common TF denominators, particularly for the IRF and KLF/SP TF family, a noticeable number of TFs were uniquely regulated along the trajectories. While *Irf1* and *Klf4*/*9*/*13* emerged for CD34^+^ SCs, *Irf3* and *Klf6*/*7*/*11* were solely identified for FRCs (Fig. 5D, Supplementary Fig. 11A, B), suggesting dissimilar modulatory paths during their development.

In summary, together with a large number of shared TFs, the observed TFBS enrichment patterns suggest that non-endothelial SCs contain a location-dependent epigenomic landscape, allowing distinct TF regulation in pLN and mLN. By combining branch-point-based differential expression together with identification of dynamically co-regulated gene networks, we were able to identify TFs that are potentially decisive in postnatally shaping the differentiation of CD34^+^ SCs or FRCs.

### Members of the IRF TF family contribute to the differentiation of CD34^+^ SCs and FRCs

By combining orthogonal analyses of TFBS enrichment and gene expression along mLN development, we were able to identify a concise assembly of TFs that are putatively involved in the differentiation of non-endothelial SC subsets. We therefore hypothesized that these TFs should be able to drive the differentiation from multipotent progenitor cells towards a cell-type or state-specification resembling ex vivo profiled SC subsets.

**Fig. 6:**
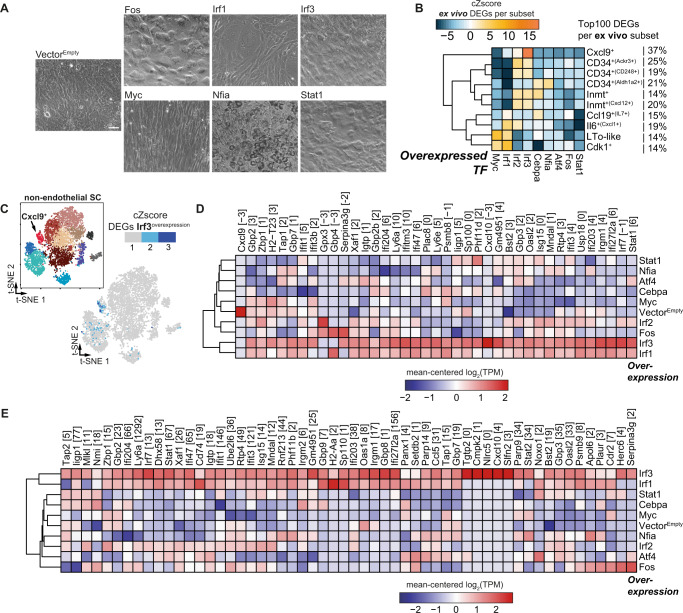
*Irf3* promotes the differentiation of mesenchymal stem cells towards a Cxcl9^+^ FRC molecular phenotype. Murine C3H10T1/2 were lentivirally transduced and puromycin-selected for stable vector integration. TF expression was doxycycline-induced and maintained for 12 days. Subsequently, BRB-seq was performed on the induced cells (3-4 replicates per condition from two independent experiments). **A** Representative phase contrast images of morphological phenotypes subsequent to overexpression-driven differentiation (scale bar = 30 µm). **B** The heatmap depicts the cumulative Z-score (cZscore) of DEGs, compared to Vector^Empty^, at “TF overexpression” in the C3H10T1/2 cell line among the Top 100 DEGs per LN subset (Supplementary Data 2). The percentage indicates the proportion of DEGs that were identified per subset (see Fig. 1, Supplementary Data 2) and the DEGs identified with”TF overexpression” in C3H10T1/2. **C** The cumulative Z-score on a per cell basis of the significant DEGs due to *Irf3* overexpression superimposed on the scRNA-seq developmental map. **D** The heatmap depicts all detected DEGs from the Cxcl9^+^ cluster for the tested (overexpressed) TFs. **E** The heatmap depicts all detected DEGs from a previously published pLN Cxcl9^+^ cluster signature^12^ for the indicated overexpressed TFs. cZscore cumulative Z-score, DEG differentially expressed gene, TF transcription factor, FRC fibroblastic reticular stromal cell, SC stromal cell, TPM transcripts per kilobase of exon per million reads. Source data are provided in a Source data file.

To this end, we utilized a well-established TF overexpression model based on lentiviral integration, puromycin selection and timed doxycycline-driven overexpression^49^ in a murine C3H10T1/2 mesenchymal stem cell like (MSC) line^50^. We focused on TFs that were either identified for both CD34^+^ SCs and FRCs (e.g., *Atf4, Nfia, Irf1*), only for CD34^+^ SCs (e.g., *Irf2, Cebpa*), or only for FRCs (e.g., *Irf3*, *Stat1*). We also included *Myc*, as LTos have myofibroblastic features^9^, and *Fos* as it is widely expressed across all non-endothelial SC subsets (Supplementary Fig. 1E)^11,12^. We initially assessed cellular morphological changes of differentiated MSCs and observed a broad range of morphologies, including the expected upregulation of lipid droplet formation upon *Nfia* overexpression^51^ and distinctive fibroblastic features for the majority of overexpressed TFs, including *Irf1, Irf3,* and *Myc* (Fig. 6A). We then assessed the gene expression profile of induced MSCs by performing bulk 3’RNA-seq (BRB-seq)^52^ at the terminal differentiation time point, twelve days post doxycycline-driven overexpression. To compare the extent of matching expression signatures for each of the overexpressed TFs with the endogenous subset-specific signatures, we calculated the cumulative Z-score (cZscore) for the Top 100 DEGs for each of the endogenously identified SC subsets (Supplementary Data 2). As expected, *Myc* overexpression drove an LTo-like expression signature, consistent with a myofibroblastic origin, but interestingly also induced a strong overlap with the molecular phenotype of the Cdk1^+^ cluster (Fig. 6B)^9^. The most striking overlap was observed for *Irf3* overexpression, supporting the expression signature of DEGs identified for Cxcl9^+^ FRCs (Fig. 6B), which was further corroborated on a per cell basis when calculating the cZscore from the *Irf3-*driven overexpression (Fig. 6C). Furthermore, *Irf3* overexpression supported the upregulation of signature genes of the Cxcl9^+^ FRC subset, including several interferon-induced proteins (e.g., *Ifit1, Ifit3, Ifit3b*) and anti-viral response elements (e.g., *Isg15, Oasl2*) above the expression levels detected for any of the other overexpressed TFs including *Irf1* and *Irf2* (Fig. 6D). In addition, the similarity between the *Irf3*-driven molecular phenotype and Cxcl9^+^ FRCs was further substantiated upon comparison to the Cxcl9^+^ FRC signature published by Rodda et al^12^. (Fig. 6E). Besides *Irf3*, *Irf1* overexpression also elicited a gene expression profile resembling Cxcl9^+^ FRCs (Fig. 6B, D, E). Since one of the characteristic biological features of Cxcl9^+^ FRCs is to mitigate effective migration of CXCR3^+^ cells during anti-viral immune responses, both *Irf1* and *Irf3* overexpression should promote an anti-viral state in MSCs. Indeed, we observed a substantial overlap of the *Irf3* overexpression signature with interferon response signatures^53,54^ in Cxcl9^+^ FRCs (Supplementary Fig. 12).

In summary, by overexpressing a selected set of delineated TFs, we validated *Irf3* and to a lesser degree *Irf1* to distinctly promote the development from MSCs to a cell phenotype that molecularly resembles Cxcl9^+^ FRCs.

### *Irf3* deficiency leads to a proportional reduction of Ccl19^+^ and Cxcl9^+^ FRCs

**Fig. 7:**
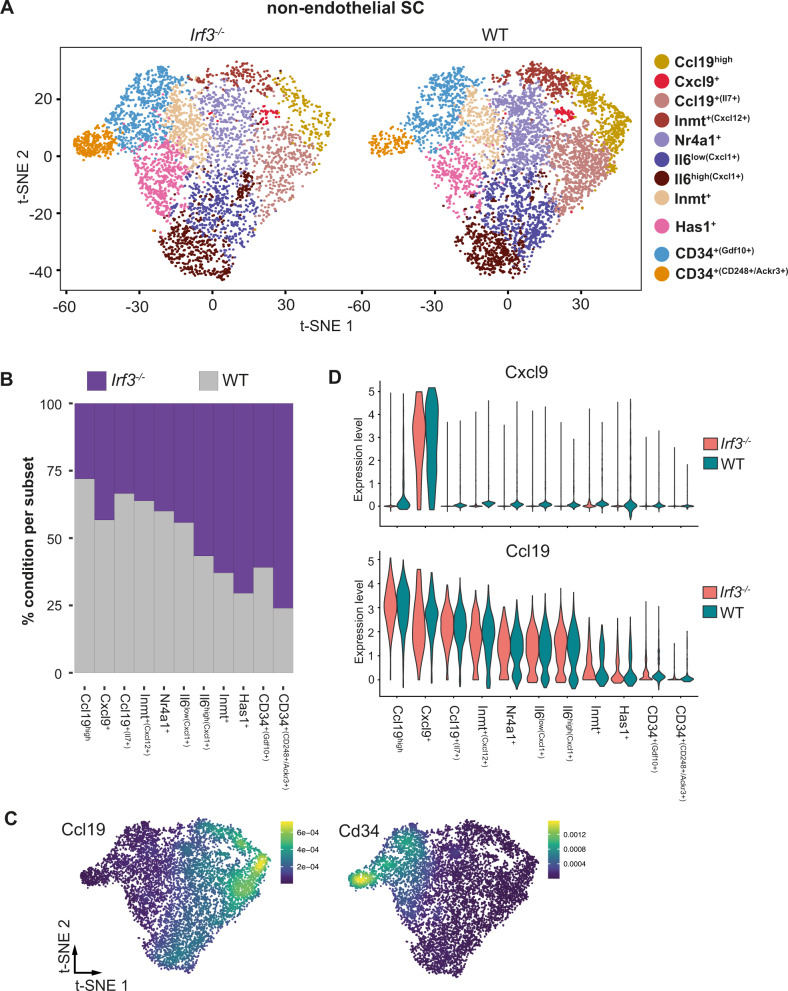
*Irf3* deficiency leads to a shift of the SC subset composition marked by a reduction of Cxcl9^+^ and Ccl19^+^ FRC subsets. CD45^-^CD24^-^ cells were isolated from mLNs and pLNs of 9–13 weeks old Irf3^−/−^ and WT male mice, and subjected to scRNA-seq analysis. One replicate was performed per condition. Non-endothelial SCs were identified as non-LECs, non-BECs, and non-PvCs. **A** t-SNE plot of integrated pLNSCs and mLNSCs showing cluster segregation of non-endothelial SC subsets between Irf3^−/−^ mice and WT controls. **B** Bar graph showing the proportion of cells within each subset normalized to total cell number belonging to either Irf3^−/−^ or WT condition. **C** Density plots depicting the expression of *Ccl19* (upper) and *Cd34* (lower) across the combined t-SNE of all non-endothelial SCs. **D** Violin plots depicting gene expression levels of *Ccl19* (upper) and *Cxcl9* (lower) across clusters between Irf3^−/−^ mice (red) and WT controls (green). BEC blood endothelial cell, FRC fibroblastic reticular stromal cell, LEC lymphatic endothelial cell, mLN mesenteric lymph node, pLN peripheral skin-draining lymph node, PvC perivascular cell, SC stromal cell, WT wild type.

Having demonstrated that *Irf3* overexpression results in a gene expression signature resembling Cxcl9^+^ FRCs, we next investigated the impact of *Irf3* on the development and phenotype of Cxcl9^+^ FRCs in vivo. To this end, CD45^-^CD24^-^ cells were FACS-purified from mLNs and pLNs of adult Irf3^−/−^ mice or WT controls. It is important to note that *Irf3* deficiency did not affect the overall frequency and absolute cell number of FRCs in pLN and mLN (Supplementary Fig. 13). Next, FACS-purified CD45^-^CD24^-^ cells were subjected to scRNA-seq analysis. Subsequent to initial quality control, 18,048 cells were processed and after exclusion of endothelial cells and perivascular cells, 11,040 non-endothelial SCs cells were re-embedded for further downstream analysis. We identified eleven transcriptional clusters based on DEGs and previously published signatures^11,12^, termed Ccl19^high^ FRC, Cxcl9^+^ FRC, Ccl19^+(Il7+)^ FRC, Inmt^+(Cxcl12+)^ FRC, Nr4a1^+^ FRC, Il6^low(Cxcl1+)^ FRC, Il6^high(Cxcl1+)^ FRC, Inmt^+^ FRC, Has1^+^ SC, CD34^+(Gdf10+)^ SC, and CD34^+(CD248+/Ackr3+)^ SC (Fig. 7A). Global inspection of the data revealed that all SC subsets, including Cxcl9^+^ FRCs, were present in both Irf3^−/−^ and WT mice, suggesting that Irf3 is not essentially required for the development of Cxcl9^+^ FRCs in vivo. However, the size of the Cxcl9^+^ FRC subset was proportionally reduced in Irf3^−/−^ mice when compared to WT controls, and this compositional shift was also observed for other SC subsets (Fig. 7A, B). While Ccl19^+^ FRC subsets were reduced, CD34^+^ SC subsets were over-represented when Irf3^−/−^ mice were compared to WT controls (Fig. 7A–C). It is important to note that the shift of the SC subset composition is not a consequence of a differential regulation of *Cxcl9* and *Ccl19* between Irf3^−/−^ and WT mice as both genes were equally expressed between both genotypes on a per subset level (Fig. 7D). When assessing general differential expression, 355 upregulated DEGs (log2FC: 0.2, padj: 0.05) were identified between Irf3^−/−^ mice and WT controls on a per subset basis, which yielded no immunologically relevant enriched GO terms.

Thus, by analyzing the non-endothelial SC compartment in LNs of Irf3^−/−^ mice we could demonstrate that Irf3 contributes to the equilibrium of SC subsets, albeit having only a minor impact on the molecular phenotype of fully differentiated SC subsets under steady-state conditions.

## Discussion

In our study, we set out to profile the mLN along postnatal development using scRNA-seq profiling, map the epigenomic landscape of the non-endothelial SC compartment of both skin- and gut-draining LNs under GF and SPF conditions and identify TFs being important for the development of individual SC subsets. We increased our understanding of three key aspects of the non-endothelial SC compartment of LNs: (1) bifuractional postnatal segregation of mesenchymal progenitors is driving the expansion of the developing LN; (2) LNSCs are marked by a tissue-specific, microbiota-independent epigenomic landscape; and (3) the Cxcl9^+^ FRC subset is controlled partly by Irf3.

We initially set out to map the postnatal mLN non-endothelial SC development on a single-cell level. scRNA-seq profiling along postnatal development identified two distinct subsets of putative proliferating progenitors that harbor the potential to yield either FRCs or CD34^+^ SCs. The surprising observation of distinct postnatal SC subsets with progenitor potential extends the perception that Cxcl13^+^ LTos are the sole subset that contributes to the expanding non-endothelial SC pool^8,23,32,55^. Although we cannot formally exclude that Cdk1^+(CD34+)^ cells originate from Cxcl13^+^ LTos and did not experimentally assess the Cxcl13^+^ LTo and Cdk1^+(Cxcl13+)^ progenitor-progeny relationship, the inferred differentiation trajectories, underscored by the distinct gene expression profiles of Cdk1^+(Cxcl13+)^ and Cdk1^+(CD34+)^ cells, support the notion that these subsets give rise to either FRCs or CD34^+^ SCs. It is conceivable that Cdk1^+(Cxcl13+)^ cells within mLNs have already established a distinct branch at birth, suggesting that a proliferating subset of commonly identified Cxcl13^+^ LTos is contributing to the postnatal expansion of the LN by transitioning through the Cdk1^+(Cxcl13+)^ state. In contrast, *Cd34* is expressed in three different locations in the neonatal mLN parenchyma: the capsule, the medulla and the adventitia of major vessels^10,12^. We noted that large vessels surrounded by an adventitial layer are scarce within the postnatal mLN, thus making it unlikely that the majority of CD34^+^ cells are of adventitial origin, which is mirrored by the distinct subset-specific gene expression profiles. However, it is important to note that trajectory analysis is predominantly an indicator for lineage relationships underscored by the misassignment of a minority of cells during cluster identification. To truly delineate the contribution of different non-endothelial SC progenitor pools, a spatio-temporal mapping of developing mLNs at pre- and postnatal stages in combination with SC fate-mapping models would be required. Recently, using scRNA-seq, in silico approaches and genetic lineage tracing Buechler et al. have identified Dpt^+^Pi16^+^CD34^+^ cells as common fibroblast progenitors across tissues, serving as a reservoir for specialized fibroblasts^56^. However, in that study SCs from mLN were not included and our own findings do not support the existence of Dpt^+^Pi16^+^CD34^+^ progenitors among LNSC. Most importantly, LTo-like cluster cells lacked expression of *Dpt* and *Pi16*, while *Dpt* was expressed rather broadly across most other mLN non-endothelial SC subsets (see Fig. 1E). Although in accordance with Buechler et al. some CD34^+^ SC subsets were Dpt^+^Pi16^+^, the majority of Cdk1^+^ cluster cells were Pi16^-^ and only a few Cdk1^+(CD34+)^ cells expressed *Dpt*, suggesting that *Dpt* and *Pi16* do not define progenitors among LNSCs.

LNSCs have long been recognized as key structural organizers, but are also increasingly perceived as effective modulators of immune responses^8,23^, even in a location-specific manner^11,21,22^. However, whether tissue-specific immunomodulatory properties of the non-endothelial SC compartment are retained in the epigenomic landscape is so far understudied^25^. Importantly, when we compared gene expression, chromatin accessibility, and DNA methylation using scRNA-seq, ATAC-seq and WGBS, respectively, we observed striking differences between skin- and gut-draining LNs, yet we could exclude a key contribution by microbial colonization. Although several studies have shown that the microbiota can impinge on the epigenome of the host’s adaptive immune system^57^, its influence does not seem to extend to the SCs of the gut-draining mLNs. This finding was surprising, as we had previously demonstrated that mLNs from GF mice lose their high Treg-inducing capacity upon transplantation into a skin-draining site, while mLNs from SPF-housed mice persistently maintained their immunomodulatory functions^11,22^. We would like to point out that the sorting of CD45^-^CD31^-^Pdpn^+^ non-endothelial SCs, which was also applied for the epigenomic profiling in the present study, yields a heterogeneous cell population that could very well mask epigenomic modifications on a per subset basis. Hence, future studies should consider to at least distinguish between the two dominant SC types in the mLN, namely FRCs and CD34^+^ SCs.

The postnatal scRNA-seq profiling in combination with the epigenetic TF footprint enabled us to identify TFs that contribute to the differentiation of distinct subsets of non-endothelial SC in adult mLNs. The identfication of those TFs enables targeted in vivo and in vitro verification of the determinants that drive the postnatal development of non-endothelial SCs. As none of the canonical markers for distinct subsets of the CD34^+^ SCs or FRCs trajectory are uniquely expressed on a per subset basis, utilization of conditional knockout mouse models in conjunction with CD34- or Ccl19-driven Cre expression would target larger and less fine-grained subsets of the LNSC compartment. We therefore opted for an in vitro TF overexpression model using a MSC line, which enabled us to screen multiple TFs and avoided the requirement to utilize neonatal progenitor cells, which are scarce and can so far not be reliably isolated due to the lack of canonical markers. The majority of overexpressed TFs did not induce differentiation towards a phenotypical profile that resembles ex vivo SCs, although a substantial number of TFs supported fibroblastic morphology. While the utilized C3H10T1/2 cell line is pre-disposed towards an adipogenic, osteogenic, myogenic or chondrogenic differentiation^58^, we did observe that overexpression of TFs from the IRF family induced a fibroblastic phenotype, likely due to the embryonic origin and thus multi-potency of the utilized MSCs^59^. In general, IFN signaling plays an important role in fibroblasts and Irf expression has been observed in stroma of multiple organs^24,60^. Particularly striking was the observation that *Irf3* and to a lesser extend *Irf1* drove the in vitro differentiation of cells towards a molecular phenotype that resembles Cxcl9^+^ FRCs, while *Irf2* did not appear to support this specific molecular phenotype. Within LNs, expression of *Cxcl9* by Cxcl9^+^ FRCs is critically required to establish the chemotactic driving forces that enable the initial detection of pathogenic incursions^20^. The distinct positioning of the proportionally small cellular compartment of Cxcl9^+^ FRCs in close proximity to lymphatic entry points enables rapid detection of tissue-originated pathogenic incursions, and subsequently accumulation of memory CD8^+^ T cells^12,20^. Importantly, Cxcl9^+^ FRCs are conserved across different LNs draining distinct tissues^11,12^. *Irf3* overexpression established robust upregulation of various interferon response genes, in line with the upregulation of *Stat1*, but did not result in an elevated expression of *Cxcl9*, a process which could rely either on additional stimulators such as IFNβ or the requirement for combinatorial TF expression^61,62^. Surprisingly, the Cxcl9^+^ FRCs could neither be annotated to the CD34^+^ SC nor the FRC developmental trajectories and showed varying expression for *Ccl19*, *Ly6c1,* and *Bst1*, indicating that Cxcl9^+^ FRCs are still heterogeneous themselves.

The scRNA-seq analysis of the non-endothelial SC compartment in LNs of Irf3^−/−^ mice revealed that Irf3 is not essentially required for the development and phenotype of Cxcl9^+^ FRCs as only a slightly reduced proportion of Cxcl9^+^ FRCs was observed in Irf3^−/−^ mice when compared to WT controls. This observation indicated that other developmental mediators can compensate for *Irf3* deficiency. Instead, it turned out that Irf3 acts more broadly since its deficiency resulted in a compositional shift across all SC subsets under steady-state conditions assessed in the present study. This finding together with the previously made observation that *Irf3* is constitutively expressed in LNSCs^24,41^ suggests that *Irf3* has multiple functional roles in various non-endothelial SC subsets, and that basal *Irf3* expression levels are likely important for the maintenance of the Ccl19^+^ FRC compartment under steady-state conditions. Yet, it is also conceivable that upon viral infection Cxcl9^+^ FRCs represent LNSCs that can acquire anti-viral properties, which was simulated by *Irf3* and *Irf1* overexpression in the MSC cell line. Under inflammatory conditions, high levels of IFN could then amplify the Cxcl9^+^ FRC molecular phenotype via enhanced *Irf3* expression. While the increase of Cxcl9^+^ FRCs upon viral infection is well documented^20,63^, it remains unclear if Cxcl9^+^ FRCs represent a unique subset that is rapidly expanded upon infection or a transient state which Ccl19^+^ FRCs can enter during inflammatory immune responses. Furthermore, the impact of *Irf3* deficiency on the FRC compartment under inflammatory conditions needs to be determined. It is noteworthy that *Irf2* overexpression did not induce the Cxcl9^+^ FRC molecular phenotype, likely because Irf2 mostly confers anti-inflammatory effects and functions as a repressor of Irf1-mediated signaling^64,65^. Hence, it will be critical to investigate the effect of individual members of the IRF family and their combinatorial effects in more detail in future studies, particularly during inflammation and infection.

We anticipate that future spatial- and subset-specific transcriptional and epigenomic profiling applied to non-endothelial SCs will further identify defining features of the immunomodulatory potential of the heterogeneous non-endothelial SCs of mLNs. The transcriptomic analysis of the postnatally developing mLN together with the epigenomic profiling across skin- and gut-draining LNs summarized in the present study can function as a valuable resource to further dissect LNSC expansion and immune modulation, and paving the way to identify targets to tissue-specifically modulate immune responses by leveraging the embedded SC immune memory and unique functional properties.

## Methods

### Mice

CD90.1 (BALB/c), Foxp3^hCD2^ (C57BL/6J), and Irf3^−/−^ (C57BL/6J)^66^ mice were bred and kept under SPF conditions in isolated ventilated cages at the Helmholtz Centre for Infection Research (Braunschweig, Germany). GF mice (BALB/c) were generated at Hannover Medical School (Hannover, Germany) by cesarean section and maintained either in plastic film isolators or in static micro-isolators at Hannover Medical School or the Helmholtz Centre for Infection Research (Braunschweig, Germany). If not stated otherwise, female mice of defined ages were used. In all experiments, age- and gender-matched mice were used. All mice were housed and handled in accordance with good animal practice as defined by FELASA and the national animal welfare body GV-SOLAS, and water and food were supplied ad libitum.

### Antibodies

Fluorochrom-conjugated anti-CD11b (clone M1/70, AF700, eBioscience Cat. #56-0112-82, 1:800), anti-CD24 (clone M1/69, APC, BioLegend Cat. #101814, 1:800), anti-CD31 (clone 390, PE-Cy7, BioLegend Cat. #102418, 1:1000), anti-CD45 (clone 30-F11, APC, BioLegend Cat. #103112, 1:400), anti-CD45 (clone 30-F11, HV510, BD Cat. #561487, 1:400), anti-Pdpn (clone 8.1.1, PE, BioLegend Cat. #127408, 1:1000), anti-Ly6G (clone 1A8, PE-Cy7, BD Cat. #560601, 1:1000), and Ter119 (clone Ly-76, APC, Biolegend Cat. #116212, 1:500) were utilized in this study. For FcγR blocking, anti-CD16/CD32 (clone 2.4G2, BioXcell, Cat. #BE0008, 1:100) was used.

### RNAscope FISH

Triple hybridizations were carried out using the RNAscope Multiplex Fluorescent Detection Kit v2 (Advanced Cell Diagnostics, Cat. #320871 #300041 #323100 #310023 #310018) in combination with the corresponding 4-Plex Ancillary Kit (Advanced Cell Diagnostics, Cat #323120 #321831). The following target probes were used: Mm-Cdk1 (Cat. #476081, targeting bp 58-1159), Mm-Cxcl13 (Cat. #406311-C2, targeting bp 2-1143), Mm-Ccl19 (Cat. #432881-C2, targeting bp 5-712), Mm-CD34 (Cat. #319161-C3, targeting bp 383-1590) and Mm-CD31(Pecam1) (Cat. #316721-C4, targeting bp 915-1827). Sample preparation and stainings were carried out according to manufacturer’s instructions. In brief, mLNs from 0-1d old SPF-housed mice were dissected and fixed in 10% neutrally buffered formaldehyde for 16–32 h at RT, washed with 1x PBS (Gibco, Cat. #14190169), and dehydrated in a series of ethanol and xylene submersions before embedding in paraffin (Merck, Cat. #76242). Formalin-fixed paraffin-embedded (FFPE) tissue blocks were stored at 4 °C. Sliced 3 µm tissue sections were continuously stored at 4 °C until RNAscope stainings were performed. FFPE sections were baked at 60 °C for 1 h, before being deparaffinized and dehydrated. Tissue sections were incubated with hydrogen peroxide for 10 min at RT before target retrieval was carried out in a steamer (Braun, Type 3216) for 15 min at >98 °C. Protease treatment was performed with Protease Plus (Advanced Cell Diagnostics, Cat. #322380) for 20 min in a humidified hybridization chamber at 40 °C. Subsequently, probes were allowed to hybridize to their targets for 2 h at 40 °C in the hybridization chamber. During the following horse radish peroxidase (HRP) based amplification process, the tyramide signal amplification (TSA)-conjugated fluorophores Opal520, Opal570, and Opal650 (Perkin Elmer, Cat. #NEL80001KT) were used to visualize target probes. Tissue sections were counter-stained with DAPI (Merck, Cat. #D9542) and mounted with ProLong Gold Antifade Mountant (Thermo Fisher Scientific, Cat. #P10144). Sections incubated with negative control probes (DapB) were stained in parallel and a mix of positive control probes (POLR2A, PPIB, UBC, Hprt) was utilized to confirm RNA integrity in each assessed tissue block. Images were acquired with an Olympus VS120 slide scanner fluorescence microscope using the VS-ASW-FL software (versions 2.9 and 2.92, Olympus). Z-stacks were acquired at ×40 or ×20 magnification and extended focus imaging (EFI) was done at ×20 magnification.

### Stromal cell isolation and flow cytometric analysis

For SC isolation, skin-draining pLNs (inguinal and axillary) or mLNs (small intestinal and colon/cecum-draining) were resected and digested in RPMI 1640 medium (Gibco, Cat. #72400021) containing 0.2 mg/ml collagenase P (Roche, Cat. #11213865001), 0.15 U/ml dispase (Roche, Cat. #4942078001) and 0.2 mg/ml DNase I (Roche, Cat. #4536282001) as described previously^67^. After digestion, cells were kept at 4 °C in PBS containing 0.2% BSA (Merck, Cat. #A2058) and 5 mM EDTA (Roth, Cat. #8043.1). CD45^-^ cells were enriched by autoMACS separation after magnetic labeling of CD45^+^ cells using anti-CD45-APC followed by anti-APC microbeads (Miltenyi Biotec, Cat. #130-090-855) or anti-CD45 Nanobeads (Biolegend, Cat. #480028). Subsequently, a small aliquot of the CD45^-^ fraction was taken for quantification of the absolute cell number (MACSQuant) and the remaining cells were stained using fluorochrome-coupled antibodies. Stained cells were either used for flow cytometric analysis (Fortessa) or to sort CD45^-^CD24^-^CD31^-^Pdpn^+^ non-endothelial SCs (Aria II, 100 μm nozzle) for RNA-seq and ATAC-seq, CD45^-^CD31^-^Ter119^-^Pdpn^+^ non-endothelial SCs (Aria II, 100 μm nozzle) for WGBS, and CD45^-^CD24^-^ non-hematopoietic cells (Aria II SORP and Aria III, 70 μm nozzle) for scRNA-seq.

### Neutrophil flow cytometric analysis

For neutrophil staining, skin-draining pLNs (inguinal and axilliary) or mLNs (small intestinal and colon/caecum draining) were isolated. Resected LNs were gently meshed through a 30 µm strainer to create single-cell suspensions. Cells were washed once with PBS containing 0.2% BSA and samples were treated with FcγR block before staining with anti-CD45, anti-CD11b, and anti-Ly6G fluorochrome-coupled antibodies. After staining, cells were resuspended in PBS containing 0.2% BSA before Precision Count Beads (BioLegend) were added to the samples according to manufacturer’s instructions. Flow cytometry analysis was carried out using a FACSymphony A5 flow cytometer and the FlowJo software (version 10.8.1; BD Biosciences) to obtain neutrophil count and frequency.

### Transfection and lentiviral packing

Reverse lentiviral transfection was performed using Lipofectamine 2000 (Thermo Fisher, Cat. #11668027) following the manufacturer’s instruction. TF-bearing lentiviral vectors^49^ and lentiviral packaging plasmids pRSV-Rev (addgeneID 12253), pMDLg/pRRE (addgeneID 12251) and pCMV-VSV-G (addgeneID 8454) were supplemented at the ratio 1:1:1:1 and incubated for 30 min at the room temperature. Prior to transfection, HEK 293T cells (ATCC Cat. No. SD-3515) were washed with PBS (Thermo Fisher, Cat. #14190169) and dissociated using 0.25% Trypsin-EDTA (Life Technologies, Cat. #25200056). Cells were resuspended in DMEM (Gibco, 41966029) 10% FBS (Gibco, Cat. #10270106) and 1% penicillin-streptomycin (Life Technologies, Cat. #15140-122) and seeded in individual wells at confluence of 95% with the transfection mix. 12 h post-transfection, fresh medium was added to attached cells and the virus-containing supernatant collected after 48 h, dead cells removed by centrifuging at 300 × *g* for 10 min and supernatant stored at −80 °C for up to 6 months.

### Lentiviral transduction

Murine C3H10T1/2 cells (ATCC Cat. No. CCL-226) were seeded 12 h prior to transduction at confluence of 10-20%. Transfection lentivirus-containing supernatant was mixed at 1:1 ratio with fresh medium and 10 µl/ml polybrene (Sigma, Cat. #TR-1003-G) and added to the plated adherent C3H10T1/2 cells. Cells were then centrifuged at 1300 g for 30 min at 37 °C, incubated with the respective lentivirus for 24 h and fresh medium was added after 24 h. After 48 h, transduced cells were selected using 2 µl/ml of puromycin (Thermo Fisher, Cat. #A1113803) for 72 h. Subsequent to puromycin selection, medium was replaced and puromycin-resistant cells permitted to recover for 48 h. Then, medium containing 2 µl/ml doxycycline was added (Sigma, Cat. #D9891-1G) to induce TF expression. Medium with doxycycline was replenished every 48 h and doxocycline treatment maintained for 12 days. Direct-zol RNA kit (Zymo Research, Cat. #R2052) was used to extract RNA according to the manufacturer’s instruction.

### Library preparation WGBS

Genomic DNA was isolated from purified CD45^-^CD31^-^Ter119^-^Pdpn^+^ non-endothelial SCs using the AllPrep DNA/RNA Micro Kit (Qiagen, Cat. #80284) according to the manufacturer’s instructions. Concentration and quality of the purified genomic DNA (gDNA) were determined by using NanoDrop (Thermo Fisher Scientific). Fragmentation of gDNA was carried out via Covaris S2 (Covaris), at duty cycle 10%, intensity 4 and 200 cycles per burst during 80 s, to obtain fragments with an average length of 300 bp. The size of the fragments was verified with Agilent Technologies 2100 Bioanalyzer. DNA sequencing libraries were generated from fragmented gDNA using the TruSeq DNA Sample Prep Kit v2 (Illumina, Cat. #15026486) according to the manufacturer’s instructions and extending the workflow by adding one additional step: Subsequent to ligation of the adapter molecules to the DNA fragments, the sample was subjected to bisulfite conversion reaction using the EZ DNA Methylation Kit (Zymo Research, Cat. #D5001). The protocol for the True Seq DNA generation was then followed. The bisulfite-converted library was amplified by performing a PCR reaction (10 cycles, 98 °C for 10 s, 63 °C for 30 s, 72 °C for 1 min) including the TruSeq primer mix and the KAPA Hifi Uracil+ Poly-merase Master Mix (Kapa Biosystems, Cat. #KK2801). The PCR product was purified and size controlled by Agilent Technologies 2100 Bioanalyzer (High Sensitivity DNA Chip). The libraries were sequenced on an Illumina HiSeq2500 sequencer using the TruSeq SBS Kit v3-HS (200 cycles, paired-end run) with an average of 2*10^8^ reads per sample. The WGBS raw and processed data have been deposited in the NCBI GEO database under accession code GSE172526.

### Library preparation ATAC-seq

CD45^-^CD24^-^CD31^-^Pdpn^+^ non-endothelial SCs were sorted by FACS into PBS containing 0.2% BSA. Cells were washed once with PBS before DNA transposition was performed with the Nextera DNA Library Prep Kit (Illumina, Cat. #FC-121-1031). Per sample, 25 µl TD, 2.5 µl TDE1 and 22 µl nuclease-free water were combined and placed at 37 °C for 3 min before 0.5 µl of 1% Digitonin (Promega, Cat. #G9441) was added to the master mix. Samples were resuspended in the transposition reaction mix and incubated for 30 min at 37 °C at 300 rpm. After transposition, DNA was purified with the MinElute PCR Purification kit (Qiagen, Cat. #28006) according to manufacturer’s instructions and eluted in 50 µl nuclease-free water. Transposed DNA fragments were pre-amplified using 10 μl transposed DNA, 10 μl nuclease-free water, 2.5 μl 25 μM custom Nextera PCR primer 1, 2.5 μl 25 μM custom Nextera PCR primer 2, 25 μl NEBNext High-Fidelity 2x PCR Master Mix (New England BioLabs, Cat. #M0541L) per reaction and amplified via a 6-cycle PCR program (1 cycle of 72 °C for 5 min, 98 °C for 30 s; 5 cycles of 98 °C for 10 s, 63 °C for 30 s, 72 °C for 1 min). The forward primer was identical for all samples 5′-AATGATACG GCGACCACCGA GATCTACACTC GTCGGCAGCGT CAGATGTG-3′, whereas the reverse primer contained distinct barcodes (example underlined) used for demultiplexing 5′-CAAGCAGAAGA CGGCATACGAG AT*TCGCCTTA* GTCTCGTGGGC TCGGAGATGT-3′^68^. The appropriate amount of further amplification cycles was determined by qPCR using 5 µl of the pre-amplified product. Final amplification was carried out with 45 μl of previously PCR amplified DNA, 39.7 μl nuclease-free water, 2.25 μl 25 μM customized Nextera PCR primer 1, 2.25 μl 25 μM customized Nextera PCR Primer 2, 0.81 μl 100x SYBR Green I and 45 μl NEBNext High-Fidelity 2x PCR Master Mix with the PCR program of 1 cycle of 98 °C for 30 s; 8–10 cycles (depending on qPCR results) of 98 °C for 10 s, 63 °C for 30 s, 72 °C for 1 min. PCR purification was carried out using the MinElute PCR Purification kit. Finally, size selection was performed with SPRIselect beads (Beckmann-Coulter, Cat. #B23317) with 1.2x for left-side and 0.55x for right-side selection according to manufacturer’s instructions. DNA quality, content, and fragment size was assessed with Agilent Technologies 2100 Bioanalyzer profiles and Qubit measurements. Libraries were sequenced on an Illumina NovaSeq6000 sequencer using 50 bp single-end reads with an average of ca. 3*10^7^ reads per sample, and quality of sequenced libraries was verified with *FastQC*. The ATAC-seq raw and processed data have been deposited in the NCBI GEO database under accession code GSE172526.

### Library preparation RNA-seq

Total RNA was extracted from FACS-sorted CD45^-^CD24^-^CD31^-^Pdpn^+^ non-endothelial SCs using the RNeasy Plus Micro Kit (Qiagen, Cat. #74034). cDNA was synthesized and amplified using template switching technology of the SMART-Seq v4 Ultra Low Input RNA Kit (Clontech Laboratories, Cat. #R400752), followed by purification using the Agencourt AMPure XP Kit (Beckman Coulter, Cat. #A63880). Library preparation was performed with Nextera XT DNA Library Prep Kit (Illumina). The Agilent Technologies 2100 Bioanalyzer was used to control quality and integrity of nucleic acids after each step. Deep sequencing was carried out on an Illumina HiSeq2500 sequencer using 50 bp single reads. Sequenced libraries were assessed for read quality using the FastQC tool. The RNA-seq raw and processed data have been deposited in the NCBI GEO database under accession code GSE172526.

### Library preparation scRNA-seq

Single CD45^-^CD24^-^ cells were sorted by FACS ARIA III (BD) and collected in PBS containing 0.04% w/v BSA at a density of 400 cells/μl. Chromium™ Controller was used for partitioning single cells into nanoliter-scale Gel Bead-In-EMulsions (GEMs) and Single Cell 3’ reagent kit v2 for reverse transcription, cDNA amplification and library construction (10xGenomics, Cat. #120236). The detailed protocol was provided by 10xGenomics. SimpliAmp Thermal Cycler was used for amplification and incubation steps (Applied Biosystems). Libraries were quantified by Qubit^TM^ 3.0 Fluometer (ThermoFisher) and quality checked using 2100 Bioanalyzer with High Sensitivity DNA kit (Agilent). Sequencing was performed in paired-end mode (2 × 75 cycles) on an Illumina NextSeq 500 sequencer to attain approximately 75,000 ± 25,000 reads per single cell. The scRNA-seq raw and processed data have been deposited in the NCBI GEO database under accession code GSE172526 and GSE106489 for D56 mLN-SPF^11^.

### Library preparation BRB-seq

3’end bulk mRNA cDNA library preparation and sequencing was performed following the BRB-seq strategy as previously described^52^. In brief, 20 ng of total RNA isolated from TF overexpression groups and corresponding controls were reverse transcribed using SuperScriptTM II Reverse Transcriptase (Lifetech, Cat. Cat. #18064014) with individual barcoded oligo-dT primers, featuring a 12-nt-long sample barcode (IDT). Double-stranded cDNA was generated by second strand synthesis via the nick translation method. To that end, a mix containing 2 μl of RNAse H (NEB, Cat. #M0297S), 1 μl of *E. coli* DNA ligase (NEB, Cat. #M0205 L), 5 μl of *E. coli* DNA Polymerase (NEB, Cat. #M0209 L), 1 μl of 10 mM dNTP (Thermo Fisher Scientific, Cat. #0181), 10 μl of 5x Second Strand Buffer (100 mM Tris, pH 6.9, [AppliChem, Cat. #A3452], 25 mM MgCl_2_ [Sigma, Cat. #M2670], 450 mM KCl [AppliChem, Cat. #A293], 0.8 mM β-NAD [Sigma, Cat. N1511], 60 mM (NH_4_)_2_SO_4_ [Fisher Scientific, Cat. #AC20587]), and 11 μl of water was added to 20 μl of ExoI-treated first-strand reaction on ice. The reaction was incubated at 16 °C for 2.5 h. Full-length double-stranded cDNA was purified with 30 μl (0.6x) of AMPure XP magnetic beads (Beckman Coulter, Cat. #A63881) and eluted in 20 μl of water. cDNA concentration was measured using Qubit, and cDNA quality was assessed using a Fragment Analyzer (Agilent). cDNA was tagmented with in-house Tn5^69^, and libraries were purified using AMPure XP magnetic beads (0.6X). The resulting libraries were profiled with a High Sensitivity NGS Fragment Analysis Kit (Advanced Analytical, Cat. #DNF-474) and measured with the Qubit dsDNA HS Assay Kit (Invitrogen, Cat. #Q32851) prior to pooling and sequencing on an Illumina NextSeq 500 sequencer utilizing a BRB-seq custom primer and the High Output v2 kit (75 cycles) (Illumina, Cat. #FC-404-2005). The sequencing configuration is as follows: Read1 21cycles / index i7 8cycles / Read2 55c. The BRB-seq raw and processed data have been deposited in the NCBI GEO database under accession code GSE172526.

### WGBS analysis

The sequenced 2 × 100 bp paired-end libraries were assessed for sufficient sequencing quality and potential adapter contamination using the programs *FastQC* (Babraham Bioinformatics, https://www.bioinformatics.babraham.ac.uk/projects/fastqc/), *trim_galore* (version 0.6.5; Babraham Bioinformatics, https://www.bioinformatics.ac.uk/projects/trim_galore/) and *cutadapt*^70^. Quality-controlled libraries have been mapped against the mouse reference genome (assembly GRCm38) using the bisulfite short read mapping software *BSMAP*^71^. Only uniquely, properly paired reads (*methratio.py* parameters: --unique, --paired, --remove-duplicate) were used to detect CpG methylation levels and coverage. CpG motifs with a minimum coverage of five mapped reads in at least two replicates of one condition served as input for methylation level smoothing and detection of DMRs using the Bioconductor package *bsseq* (version 1.16)^72^. Regions were classified as differentially methylated between two condictions if they (1) contain at least three CpG motifs with (2) a maximal distance of 300 bases, (3) a mean methylation difference of at least 0.25, and (4) all CpGs in the region have an associated t-statistic (*bsseq* function *Bsmooth.tstat*) beyond a [low,high] cutoff with low = 0.01 and high = 0.99 (parameter q = (low,high) of *bsseq* function *dmrFinder*).

### ATAC-seq analysis

Sequencing reads were mapped to mouse genome (mm10) using *STAR* (version 2.5.3a)^73^ with parameters *--runMode alignReads --outSAMtype BAM SortedByCoordinate --outFilterMultimapNmax 1* (assembly: GRCm38). Duplicates were removed using *picard MarkDuplicates* (https://broadinstitute.github.io/picard/). Peaks were called on de-duplicated bam-files using *macs2 callpeak* with the parameters *--broad -g mm -q 0.05* for each replicate^74^. Heatmaps of fragment distribution around the transcriptional start site were computed using *computeMatrix* with the *reference-point -a 3000 -b 3000* and plotted using *plotHeatmap*. Peak track per replicate were merged into one merged peak track together with 68 ENCODE-deposited ATAC-seq datasets for various mouse tissues^39^. The merged peak track of both the FACS-sorted CD45^-^CD24^-^CD31^-^Pdpn^+^ non-endothelial SC and the 68 ENCODE datasets was used to obtain counts for each region across all datasets. Normalization across all datasets was implemented via variance stabilization (DEseq2) together with quantile normalization. Correlation coefficients were computed via Pearson correlation, and the normalized data subjected to Euclidian clustering.

For all other analyses in the study, the regions identified via *macs2* (version 2.2.6) for each of the CD45^-^CD24^-^CD31^-^ Pdpn^+^ non-endothelial SC replicates were merged across all replicates into one set of regions, by combining peaks overlapping with at least one base-pair and removing peaks that overlapped with blacklisted regions^75^. Differential accessibility of raw ATAC-seq counts for each region/peak across all replicates of all samples were normalized across replicates with size factors computed with *DESeq2* (version 1.22)^76^. Pairwise comparisons were performed with *DESeq2* and DARs were called with an FDR adjusted *p*-value of less than 0.05 and a fold change (FC) of at least 2. Genomic features were identified via *getAnnotation* from *ChIPpeakANNO* (version 3.6.5)^77^. The cumulative FC of all DARs for one respective gene is represented as the mean of all the FC of all respective DARs. Transcription factor motif enrichment was computed using *homer* (version 4.9)^78^. Graphics were generated in R using *pheatmap* and *ggplot2*. Genomic tracks were visualized using IGV browser.

### RNA-seq analysis

Libraries were aligned versus the mouse reference genome assembly GRCm38 using the splice junction mapper Tophat2 v1.2.0 with default parameterization^79^. Reads aligned to annotated genes were quantified with the HTSeq (version 0.12.4)^80^ and determined read counts served as input to DESeq2^76^ for pairwise detection and quantification of differential gene expression. RPKM (reads per kilobase of exon length per million mapped reads) values were computed for each library from the raw read counts. For scatterplots and heatmaps only genes with an annotated official *Gene Symbol* were included. Gene ontology (GO) analyses were performed using the R package *TopGo* (version 2.12)^81^. The R packages *pheatmap* and *ggplot2* were used to generate heatmaps or scatterplots, respectively.

### scRNA-seq analysis

For the mLN ontogeny dataset, data were processed using Cell Ranger software (version 2.0.0) Count matrices were further processed with Seurat (version 2.3.3). All cells received an identifier which was used as common meta-data throughout the analysis including differentiation trajectories and dynamic gene regulatory networks (see below).

All cells with less than 1000 or more than 4600 detected genes per cell were filtered out. Moreover, cells with more than 4.5% reads mapping to mitochondrial genes were removed yielding 15,659 cells passing QC. After filtering, data were default normalized and the 2000 most variable genes identified. The expression levels of these genes were scaled before performing PCA. The following covariates were regressed out: number of UMIs, percent of mitochondrial reads, percent of ribosomal reads and scores for the proliferation S.Score and G2M.Score computed with *CellCycleScoring()*. t-SNE dimensionality reduction was performed using the first 12 dimensions of the PCA and resolution set to 1.1. Only clusters (non-endothelial SC) with normalized expression for *Pecam* < 1 were used for the further analysis amounting to 9323 cells which were re-embedded as described above (resolution = 1.0). The perivascular mesenchymal cell (PvMC) cluster and clusters classified as adjacent cells were excluded and the remainder cells, numbering 5658 mesenchymal cells were re-embedded as described above (resolution = 1.0). It should be noted that metabolically active FRC cluster cells express higher levels of ribosomal protein reads, while overall containing generally fewer reads when compared to all other subsets. For subsetting the Cdk1^+^ subset, 259 cells were re-embedded as described above (resolution = 0.5, dimsuse = 10). GO analysis was performed for differentially upregulated genes per cluster using *TopGO*^81^.

Differentiation trajectories were analyzed using *Monocle* (version 2.18.0)^33^. Unsupervised ordering was performed on the 5658 mesenchymal cells using *Monocle2’s DDRTree* algorithm based on genes with a mean expression >0.1. The trajectory containing all mesenchymal cells, was split based on marker gene expression *Vcam1* and *Cd34* and the annotation of cells belonging to the Cdk1^+(Cxcl13+)^ or Cdk1^+(CD34+)^ subset to distinct terminal branches. Two separate unsupervised orderings were performed as described above and are referred to as CD34^+^ SC or FRC trajectory.

Gene regulatory networks were inferred with the *dynGENIE3* algorithm^48^, where the input expression values were based on ordering the genes according to physiological age per branch for each of the two trajectories being CD34^+^ SC or FRC. The list of candidate TFs was derived from TFBS from ATAC-seq profiling or DMRs within the proximity of transcriptional start sites. For network visualization with Cytoscape (version 3.6.0)^82^, only the Top 500 links ranked by weight assigned by *dynGENIE3* were used. Node centrality and betweenness were calculated with the degree and betweenness functions from the igraph (version 1.2.2) package.

To compare the overlap of the Cxcl9^+^ FRC expression signature with IFN response genes, the overlap of the cZsore of the list of anti-viral IFN stimulated genes of mammalian leucocytes (cZscore ISG3)^53^ was computed across the respective gene expression per cluster. In addition, the overlap of the cZscore of an ISG set from the Interferome database of primary fibroblasts (Interferome 2.0^54^, search criteria: max. 6 h post stimulation with IFNβ, fold change > 2.0; *p*-val < 0.05) was computed across gene expression per cluster.

For the GF vs. SPF dataset, data were processed using Cell Ranger software (version 2.0.0). Count matrices were further processed with Seurat (version 4.0.1). pLN and mLN SPF sample data were previously published^11^ and deposited in the NCBI GEO database under accession code GSE116633. All cells with less than 750 or more than 4000 detected genes per cell were filtered out. Moreover, cells with more than 7% reads mapping to mitochondrial genes were removed yielding 18,045 cells passing QC. After filtering, data were default normalized and the 2000 most variable genes identified. Datasets were integrated and the expression levels of these genes were scaled before performing PCA. The following covariates were regressed out: number of UMIs, number of genes, percent of mitochondrial reads and percent of ribosomal reads. t-SNE dimensionality reduction was performed using the first 17 dimensions of the PCA and resolution set to 0.4. Only non-endothelial SC clusters were used for the further analysis amounting to 14,307 cells, which were re-embedded as described above (dims.use = 20, resolution = 0.8). Again, it should be noted that metabolically active FRC cluster cells express higher levels of ribosomal protein reads, while overall containing generally fewer reads when compared to all other subsets. GO analysis was performed for differentially upregulated genes per cluster using *TopGO*^81^.

For the Irf3^−/−^ dataset, data were processed using Cell Ranger software (version 6.0.0). Count matrices were further processed with Seurat (version 4.0.1). All cells with less than 750 or more than 4500 detected genes per cell were filtered out. Moreover, cells with more than 6% reads mapping to mitochondrial genes were removed yielding 18,048 cells passing QC. After filtering, data were default normalized and the 2000 most variable genes identified. Datasets were integrated and the expression levels of these genes were scaled before performing PCA. The following covariates were regressed out: number of UMIs, number of genes, percent of mitochondrial reads and percent of ribosomal reads. t-SNE dimensionality reduction was performed using the first 30 dimensions of the PCA and resolution set to 0.4. Only non-endothelial SC clusters were used for the further analysis amounting to 11,040 cells, which were re-embedded as described above (dimsuse = 13, resolution = 0.9). GO analysis was performed for differentially upregulated genes per cluster using *TopGO*^81^.

### BRB-seq analysis

After sequencing and standard Illumina library demultiplexing, the fastq-files were aligned to the mouse reference genome mm10 (GRCm38) using STAR (version 2.7.3a), excluding multiple mapped reads. Resulting BAM files were demultiplexed per sample using BRB-seqTools (version 1.4, https://github.com/DeplanckeLab/BRB-seqTools) and read-count matrices generated using HTSeq (version 0.12.4). Raw read counts were converted to transcripts per kilobase of exon per million reads values. Protein-coding genes with at least 5 reads in at least two replicates were included in the analysis. The calculated read counts were further processed with *DESeq2* for quantification of differential gene expression^76^. Genes were considered as differentially expressed at fold change >2.0 and the FDR adjusted *p*-value of <0.05.

### Statistical analysis

For most scripts written in R, we used version 3.4.1. Only for analysis of GF-SPF and Irf3^−/−^ scRNA-seq datasets, R version 4.0.5 was used. For flow cytometry experiments, GraphPad Prism (version 8.0.1) was used for statistical analysis. Each data point represents a single mouse and a two-tailed Mann–Whitney U test was used for the comparison of two groups. Flow cytometry based data is depicted as the mean ± SEM and *p*-values below a threshold of 0.05 were considered significant; *p < 0.05; **p < 0.01; ***p < 0.001; ****p < 0.0001; ns = not significant.

### Reporting summary

Further information on research design is available in the Nature Portfolio Reporting Summary linked to this article.

## Supplementary information

Supplementary Information

Description of Additional Supplementary Files

Supplementary Data 1

Supplementary Data 2

Supplementary Data 3

Supplementary Data 4

Supplementary Data 5

Reporting Summary

## Data Availability

ATAC-seq, WGBS, RNA-seq, scRNA-seq, and BRB-seq raw and processed data generated in this study have been deposited in the NCBI GEO database under accession code GSE172526. Source data are provided with this paper.

## Source data

Source Data

## Code availability

The code utilized for this study is available at: https://github.com/jpezoldt/LymphNode_StromaCell_Ontogeny.
