# Supplementary material for: Postnatal expansion of mesenteric lymph node stromal cells towards reticular and CD34+ stromal cell subsets
Source: Nat Commun. 2022 Nov 24;13:7227. doi: 10.1038/s41467-022-34868-4 (PMC9700677; doi:10.1038/s41467-022-34868-4)
Supplement: Supplementary file 1 — Supplementary Information [file 41467_2022_34868_MOESM1_ESM.pdf]

Postnatal expansion of mesenteric lymph node stromal cells towards reticular and CD34$^+$ stromal cell subsets

Joern Pezoldt[1,2,#], Carolin Wiechers[1,#], Mangge Zou[1], Maria Litovchenko[2], Marjan Biocanin[2], Michael Beckstette[1,3,4], Katarzyna Sitnik[5], Martina Palatella[1], Guido van Mierlo[2], Wanze Chen[2], Vincent Gardeux[2], Stefan Floess[1], Maria Ebel[1], Julie Russeil[2], Panagiota Arampatzi[6], Ehsan Vafardanejad[7], Antoine-Emmanuel Saliba[7], Bart Deplancke[2,#], Jochen Huehn[1,8*,#]

[1]Department Experimental Immunology, Helmholtz Centre for Infection Research, 38124 Braunschweig, Germany
[2]Laboratory of Systems Biology and Genetics, École Polytechnique Fédérale de Lausanne, 1015 Lausanne, Switzerland
[3]Department of Computational Biology for Individualised Medicine, Centre for Individualised Infection Medicine, Helmholtz Centre for Infection Research and Hannover Medical School, 30625 Hannover, Germany
[4]Genome Informatics Group, Bielefeld Institute for Bioinformatics Infrastructure, Department of Technology, Bielefeld University, 33615 Bielefeld, Germany
[5]Institute of Animal Breeding and Genetics, University of Veterinary Medicine Vienna, Vienna, Austria
[6]Core Unit Systems Medicine, University of Wuerzburg, 97080 Wuerzburg, Germany
[7]Helmholtz Institute for RNA-based Infection Research (HIRI), Helmholtz-Center for Infection Research (HZI), 97080 Würzburg, Germany
[8]Cluster of Excellence RESIST (EXC 2155), Hannover Medical School, 30625 Hannover, Germany

[#]Equally contributing authors
*Corresponding author: Jochen Huehn (jochen.huehn@helmholtz-hzi.de)

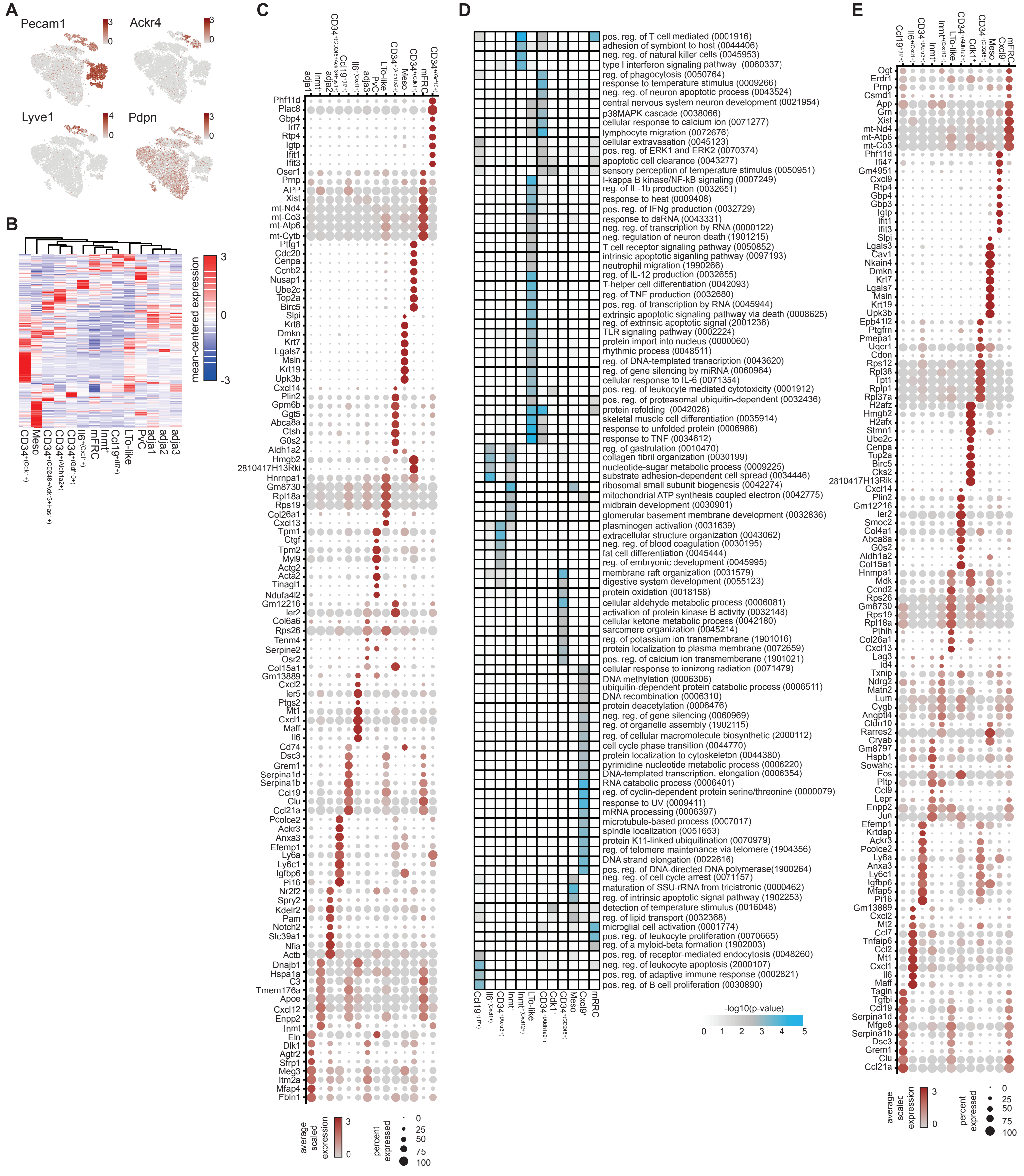

Supplementary Figure 1: The LNSC compartment can be dissected into distinct cellular subsets using scRNA-seq. *Related to Figure 1.* CD45⁻CD24⁻ cells were isolated from mLNs of day 0, 10, 24, 56 and 300 old SPF-housed mice and subjected to scRNA-seq. **(A)** t-SNE plot of merged 15,925 endothelial and non-endothelial mLN SCs. Expression of indicated genes is superimposed on the t-SNE map. **(B)** Heatmap of the top 40 DEGs across all non-endothelial SC subsets including PvC and adjacent tissue cell subsets (adja). **(C)** Expression dot plot of top 8 DEGs (selected by foldchange) per subset comparing all remaining non-LEC and non-BEC mLN SCs. **(D)** GO analysis of biological processes was performed on DEGs per non-endothelial SC subsets (enrichment analysis of GO terms calculated with Fisher´s exact test). **(E)** Expression dotplot of top 10 DEGs (selected by foldchange) per subset comparing all non-endothelial SC subsets. DEG, differentially expressed gene; GO, gene ontology; Meso, mesothelial; mLN, mesenteric lymph node; PvC, perivascular cells; SC, stromal cell; SPF, specific pathogen-free.

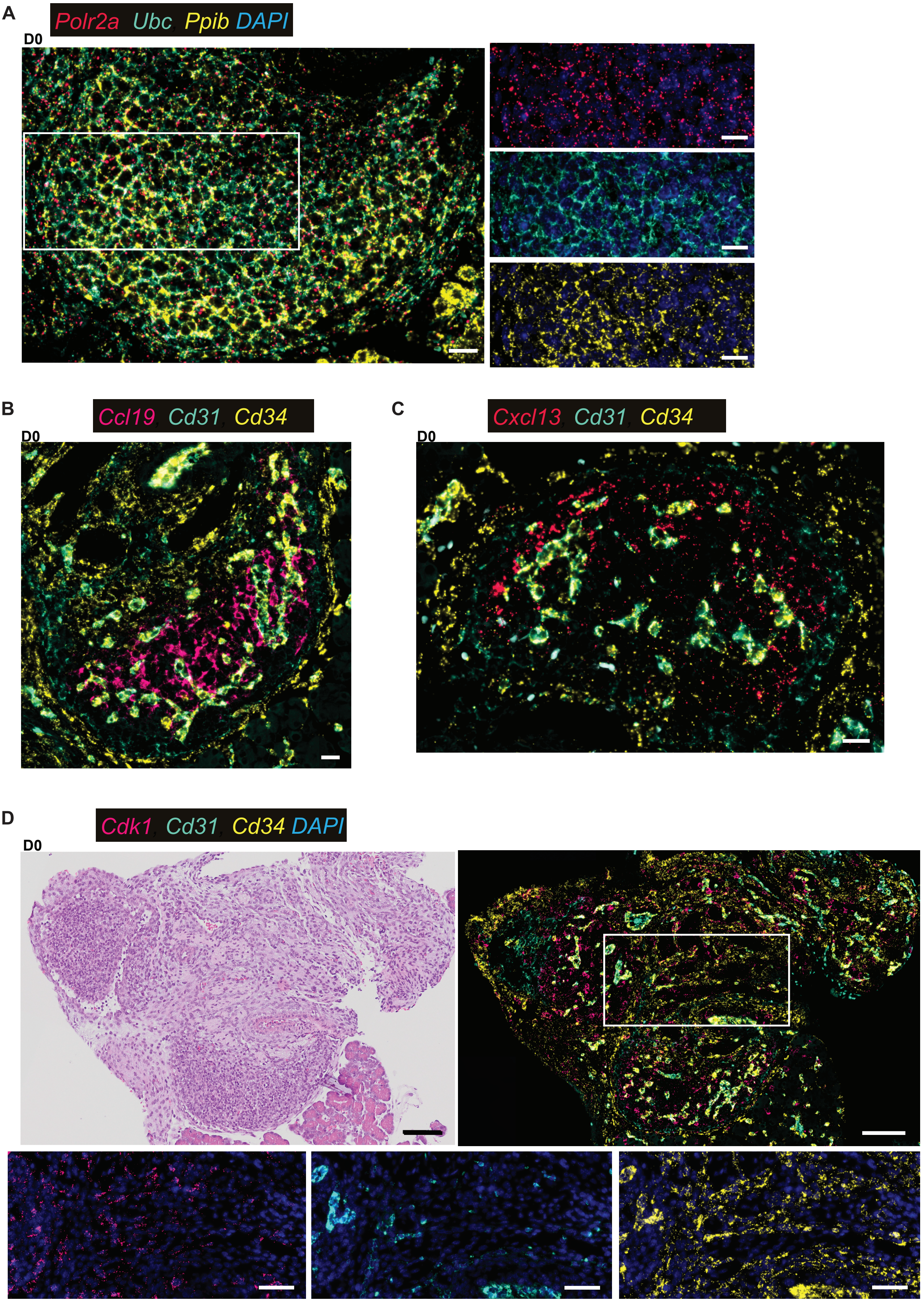

Supplementary Figure 2: Expression of key marker genes in neonatal mLNs and adjacent tissues. *Related to Figure 1.* Sections (3 µm) of early postnatal mLN (day 0, D0) were stained with indicated RNAscope probes and imaged by fluorescence microscopy. Nuclei were counter-stained with DAPI (blue). Images were subjected to linear contrast enhancement. 'White squares' indicate regions of interest. Representative tissue sections = 2-3. **(A)** Positive control probes for the genes *Polr2a*, *Ppib* and *Ubc* (scale bars = 20 µm). **(B)** Overview of mLN anlagen for RNA-probes specific for *Ccl19*, *Cd31* and *Cd34* (scale bar = 20 µm). **(C)** Overview of mLN anlagen for RNA-probes specific for *Cxcl13*, *Cd31* and *Cd34* (scale bar = 20 µm). **(D)** ***Upper left*** Haematoxylin-Eosin (HE) overview staining of D0 mLN embedded in surrounding tissue (scale bar = 100 µm). ***Upper right*** Overview of mLN anlagen for RNA-probes specific to *Cdk1*, *Cd31* and *Cd34* (scale bar = 100 µm). ***Lower*** Zoom-ins for RNA-probes specific to *Cdk1*, *Cd31* and *Cd34* (scale bar = 50 µm). HE, Haematoxylin-Eosin; mLN, mesenteric lymph node.

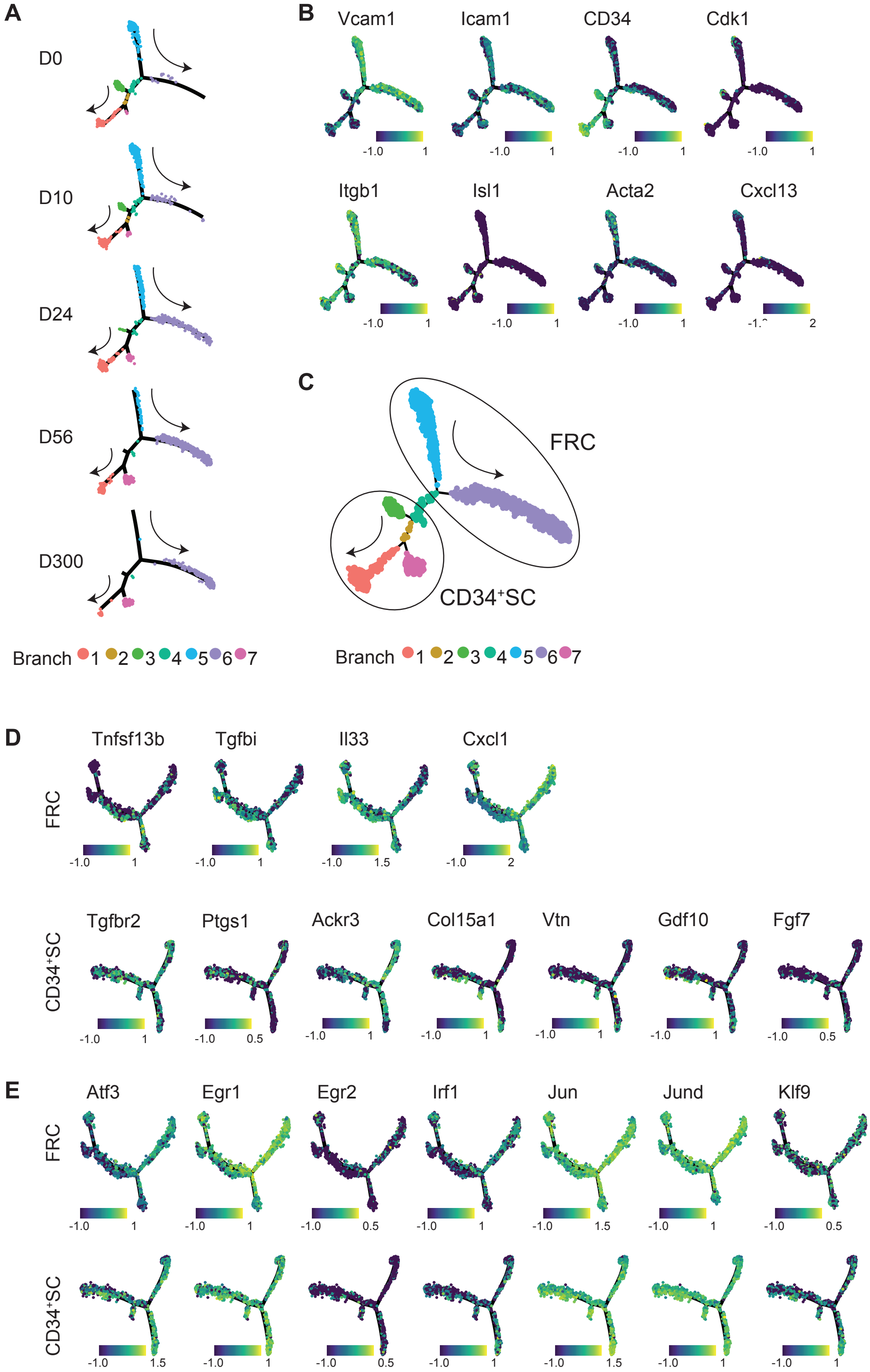

Supplementary Figure 3. Bifurcation of LNSC development. *Related to Figure 2.* $CD45^-CD24^-$ cells were isolated from mLNs of day 0, 10, 24, 56 and 300 old SPF-housed mice and subjected to scRNA-seq. Target SCs were identified as non-LECs, non-BECs and non-PvCs. **(A)** Pseudotemporal trajectory of non-endothelial SCs plotted for the indicated age. Arrows indicate increasing pseudotime. **(B)** Gene expression on the pseudotime trajectory. **(C)** Applied electronic gating for re-embedding FRCs and $CD34^+$ SCs. **(D)** Expression of selected DEGs along FRCs and $CD34^+$ SCs. **(E)** Expression of common TFs for both FRCs and $CD34^+$ SCs. DEGs, differentially expressed genes; FRC, fibroblastic reticular stromal cell; PvC, perivascular cell; SC, stromal cell; SPF, specific pathogen-free; TF, transcription factor.

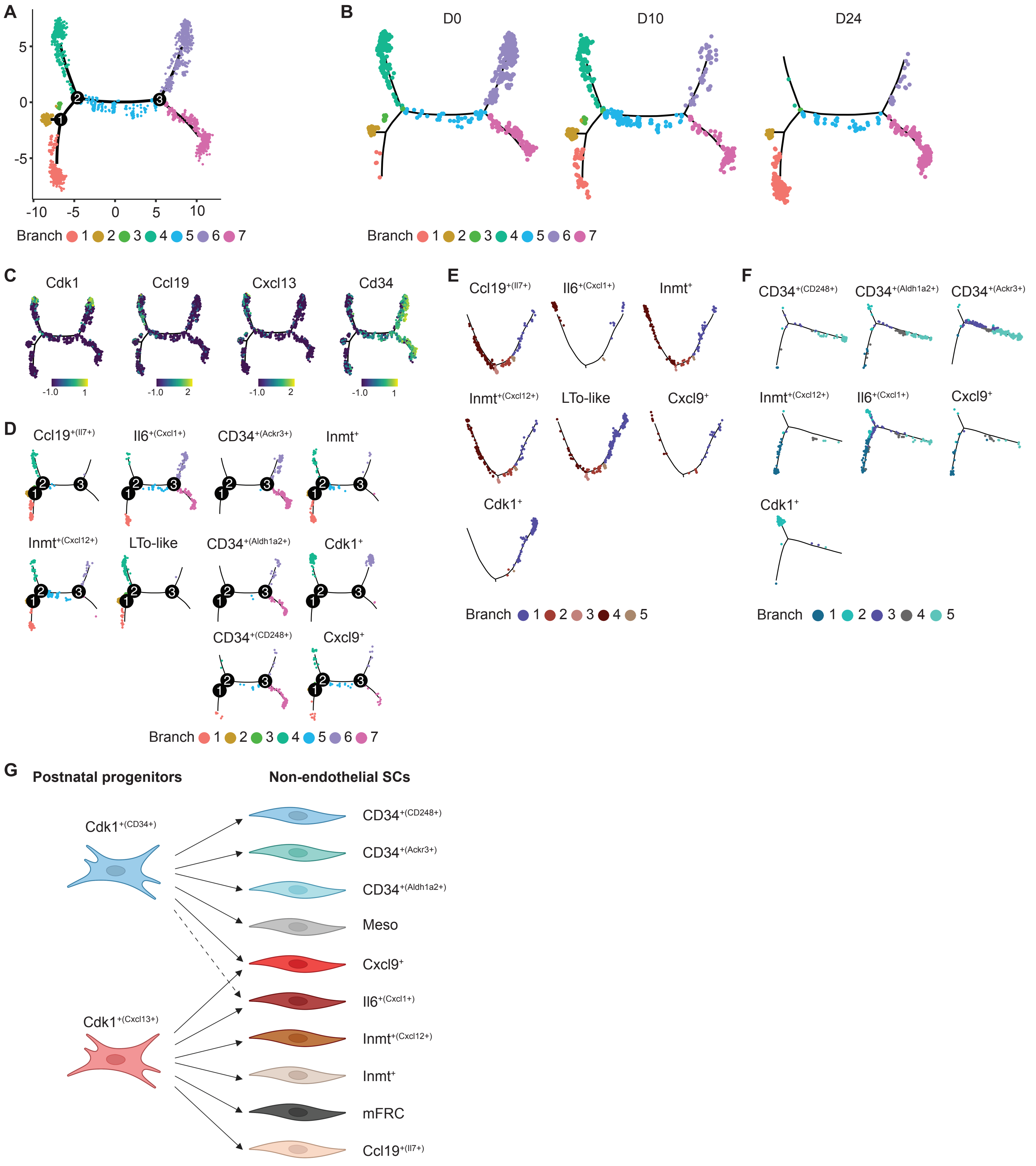

Supplementary Figure 4. Confirmation of bifurcational LNSC development in samples restricted to early life. *Related to Figure 2.* $CD45^-CD24^-$ cells were isolated from mLNs of day 0, 10 and 24 old SPF-housed mice and subjected to scRNA-seq. Target SCs were identified as non-LECs, non-BECs and non-PvCs. **(A)** Combined pseudotime ordering of FRCs and $CD34^+$ SCs. **(B)** Pseudotemporal trajectory of non-endothelial SCs plotted for the indicated age. **(C)** Gene expression on the pseudotime trajectory. **(D)** Cells per indicated non-endothelial SC subset cluster across branches superimposed on pseudotime trajectory. **(E)** Cells per indicated non-endothelial SC subset cluster across branches superimposed on FRC trajectory. **(F)** Cells per indicated non-endothelial SC subset cluster across branches superimposed on $CD34^+$ SC trajectory. **(G)** Schematic illustration of the differentiation potential of two postnatal progenitors into non-endothelial SC subsets (created with BioRender.com). DEGs, differentially expressed genes; FRC, fibroblastic reticular stromal cell; PvC, perivascular cell; SC, stromal cell; SPF, specific pathogen-free; TF, transcription factor.

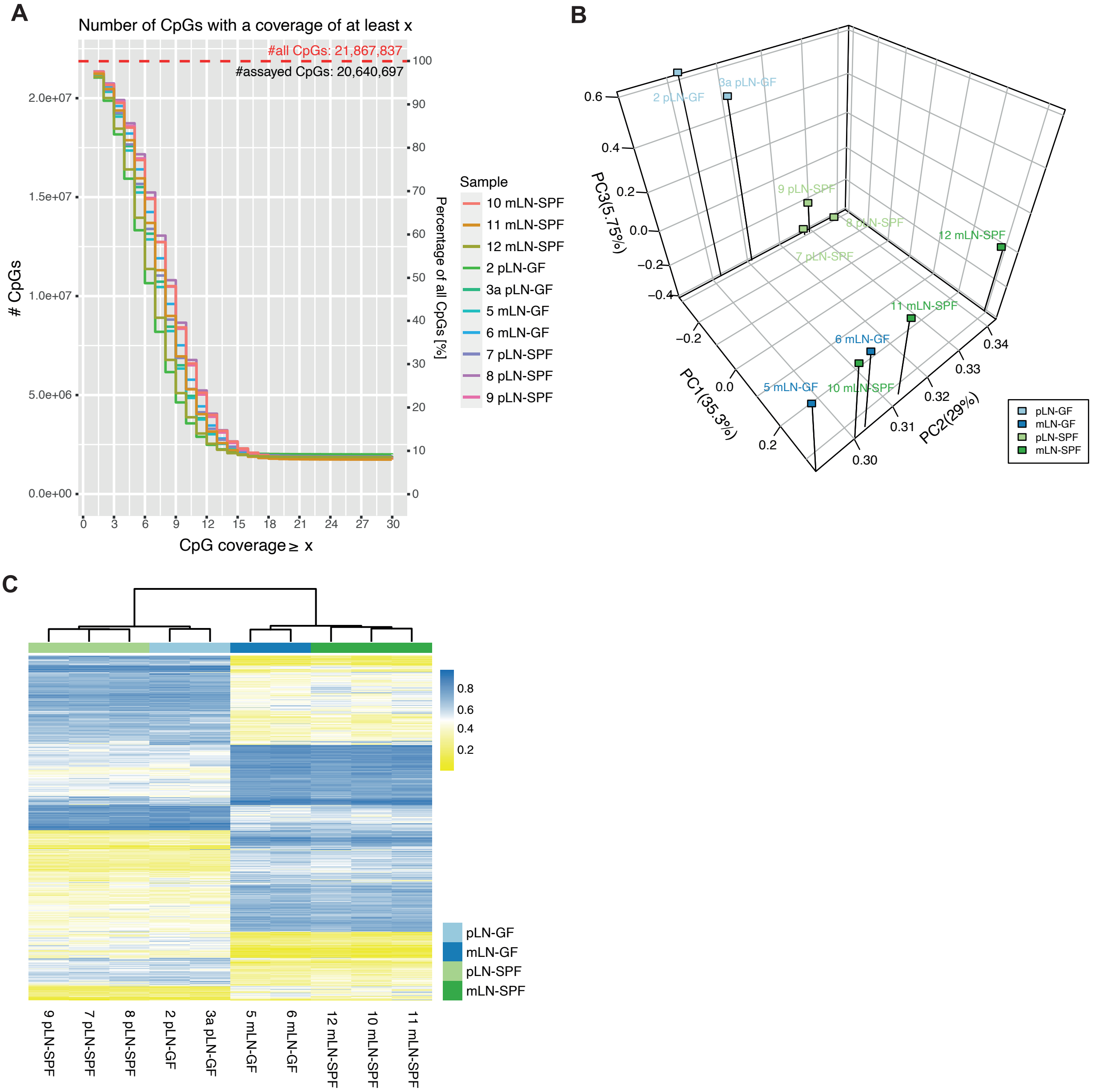

Supplementary Figure 5. PCA and hierarchical clustering of individual WGBS samples. *Related to Figure 4.* CD45$^-$CD24$^-$CD31$^-$Pdpn$^+$ SCs were isolated from mLNs and pLNs of 6-10 weeks old GF or 7-12 weeks old SPF mice. Subsequently, WGBS analysis was performed. DMRs were identified in colonization- (SPF vs. GF) and location-dependent (mLN vs. pLN) pairwise comparisons. **(A)** Coverage of assayed CpG motives for each sample. The number of all CpGs in the mouse is given in red (2.19*10^7). **(B)** PCA of scaled and centered DMR mean methylation values for all replicate samples. **(C)** Hierarchical clustering of replicate samples based on all distinct (i.e. different genomic coordinates) identified DMRs. For WGBS, 2-3 replicate samples per condition were generated. DMR, differentially methylated region; mLN, mesenteric lymph node; PCA, principal component analysis; pLN, skin-draining lymph node; SPF, specific pathogen-free; SC, stromal cell; TF, transcription factor; WGBS, whole-genome bisulfite sequencing. Source data are provided in a Source Data file.

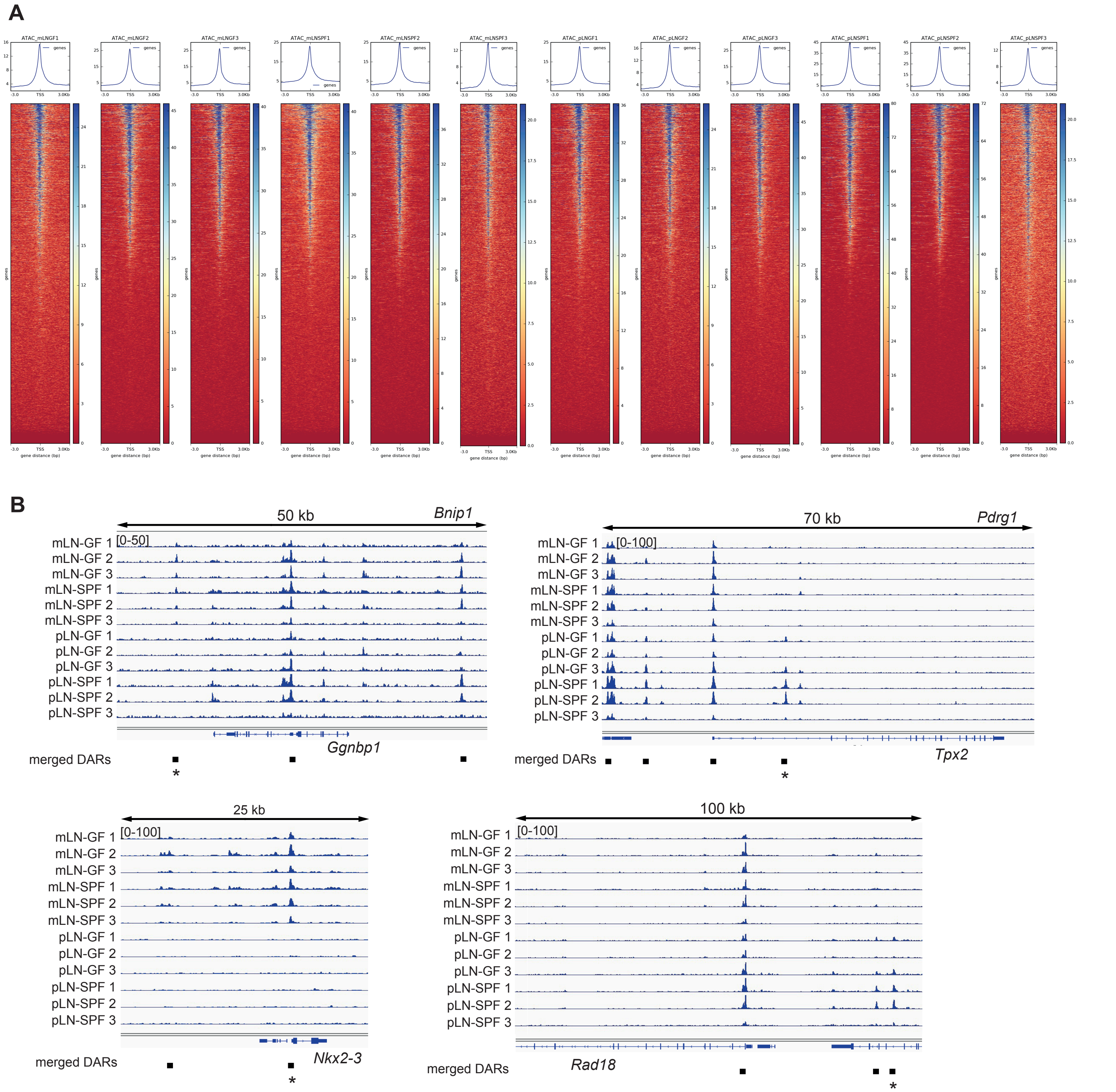

Supplementary Figure 6. TSS coverage of individual samples and ATAC-seq genome tracks. *Related to Figure 4.* $CD45^{-}CD24^{-}CD31^{-}$ $Pdpn^{+}$ SCs were isolated from mLNs and pLNs of 6-10 weeks old GF or 7-12 weeks old SPF mice. Subsequently, ATAC-seq analysis was performed. DARs were identified in colonization- (SPF vs. GF) and location-dependent (mLN vs. pLN) pairwise comparisons. **(A)** Coverage at the TSS on a per gene of ATAC-seq fragment length basis for individual replicates. Aggregated coverage at the TSS $\pm$ 3 kbp (top) and coverage of single TSS per gene (bottom). The scale is showing coverage as number of reads. **(B)** Genome tracks depicting replicates for indicated genomic regions of selected DARs among top 20 DARs between location-dependent (mLN vs. pLN) pairwise comparisons. Replicates are group-scaled and top DARs are indicated with an asterix. In case the top DAR was associated with a neighboring gene, this corresponding gene is indicated in the top right corner. For ATAC-seq, three replicate samples per condition were generated. ATAC-seq, assay for transposase accessible chromatin sequencing; DAR, differentially accessible region; FPKM, fragments per kilobase of peak per million reads; GF, germ-free; mLN, mesenteric lymph node; pLN, skin-draining lymph node; SPF, specific pathogen-free; SC, stromal cell; TSS, transcription start site. Source data are provided in a Source Data file.

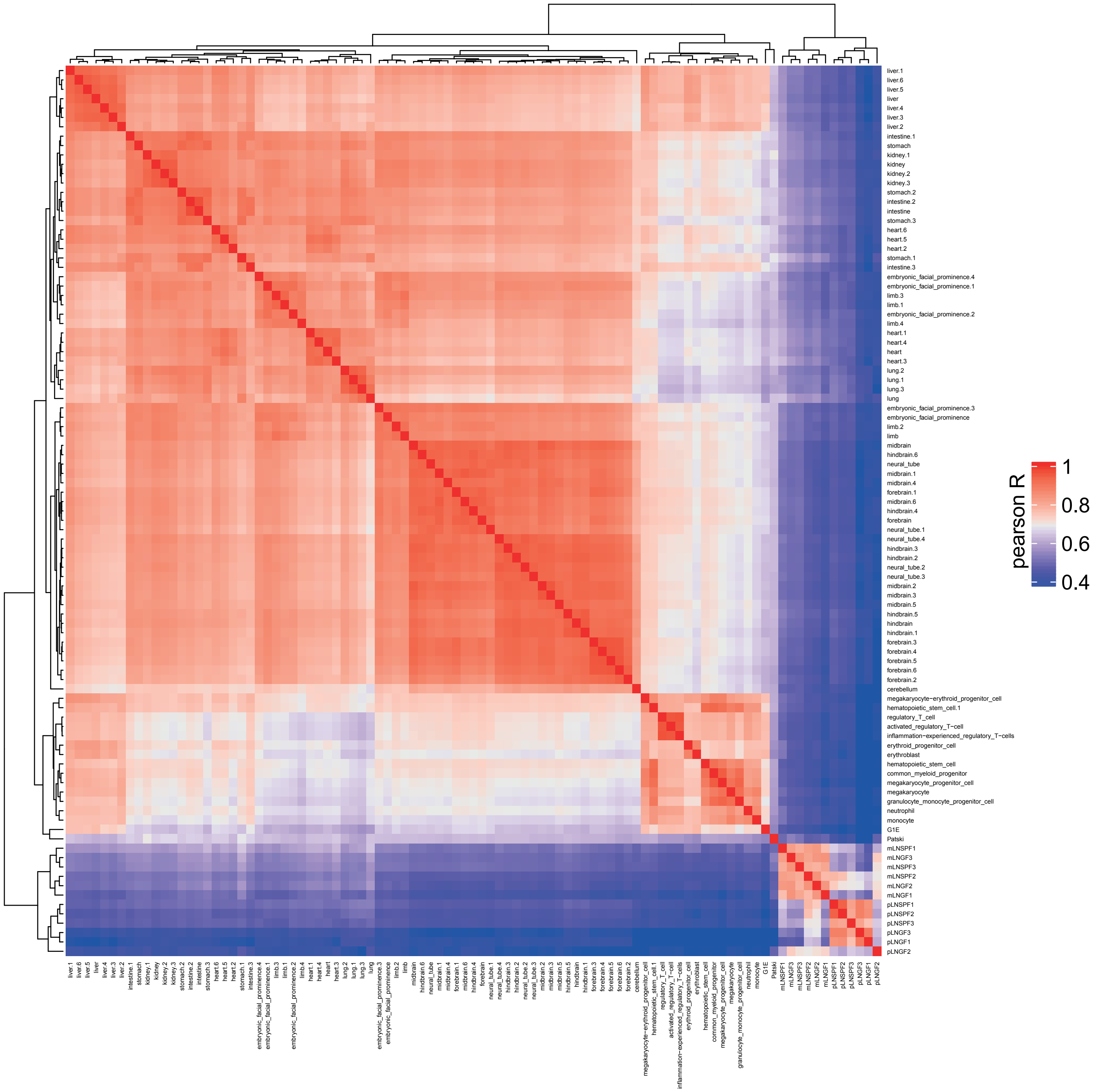

Supplementary Figure 7. Non-endothelial SC ATAC-seq profile comparison to ENCODE project datasets. *Related to Figure 4.* $CD45^{-}CD24^{-}CD31^{-}Pdpn^{+}$ non-endothelial SCs were isolated from mLNs and pLNs of 6-10 weeks old GF or 7-12 weeks old SPF mice. Subsequently, ATAC-seq analysis was performed. The merged peak track was used to obtain counts for each region. Variance stabilization (DEseq2) together with quantile normalization was utilized previous to computing pearson correlation matrices, used for Euclidian clustering. For ATAC-seq, three replicate samples per condition were generated. ATAC-seq, assay for transposase accessible chromatin sequencing; GF, germ-free; mLN, mesenteric lymph node; pLN, skin-draining lymph node; SC, stromal cell; SPF, specific pathogen-free. Source data are provided in a Source Data file.

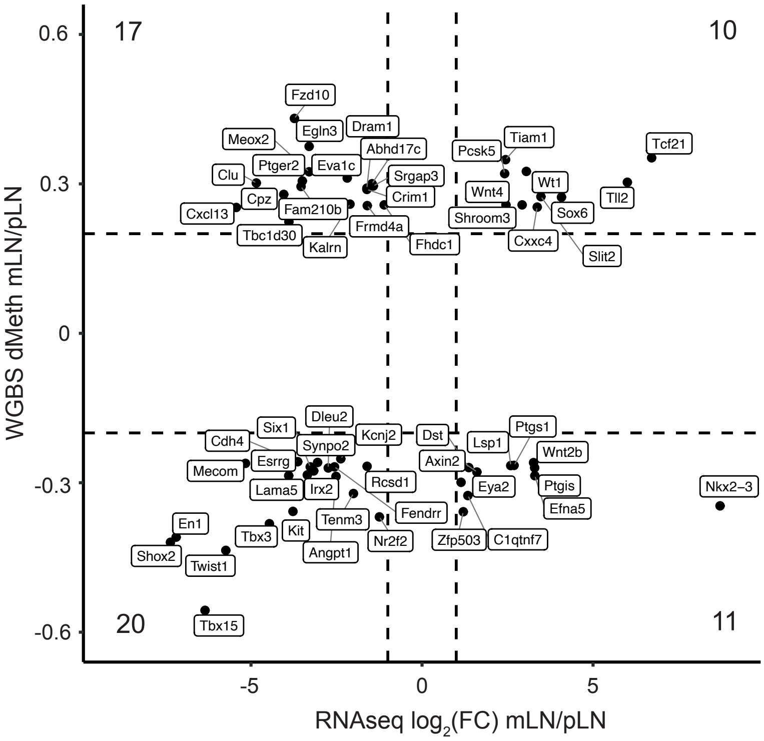

Supplementary Figure 8. Overlap of transcribed genes and methylation status. *Related to Figure 4.* $CD45^-CD24^-CD31^-Pdpn^+$ SCs were isolated from mLNs and pLNs of 7-12 weeks old SPF mice. Subsequently, WGBS and RNA-seq analyses were performed. DMRs and DEGs were identified in mLN vs. pLN pairwise comparisons. Correlation between gene expression and methylation. DMRs and DEGs were identified in mLN vs. pLN pairwise comparisons. Numbers in the scatterplot represent the number of genes with DMR and/or differential expression. On the x-axis, log2(FC) of gene expression is plotted and on the y-axis, the demethylation extent per DMRs for the comparison of mLN vs. pLN. For WGBS and RNA-seq, 2-3 and three replicate samples per condition were generated, respectively. DEG, differentially expressed gene; DMR, differentially methylated region; FC, fold change; mLN, mesenteric lymph node; pLN, skin-draining lymph node; SPF, specific pathogen-free; SC, stromal cell; WGBS, whole-genome bisulfite sequencing. Source data are provided in a Source Data file.

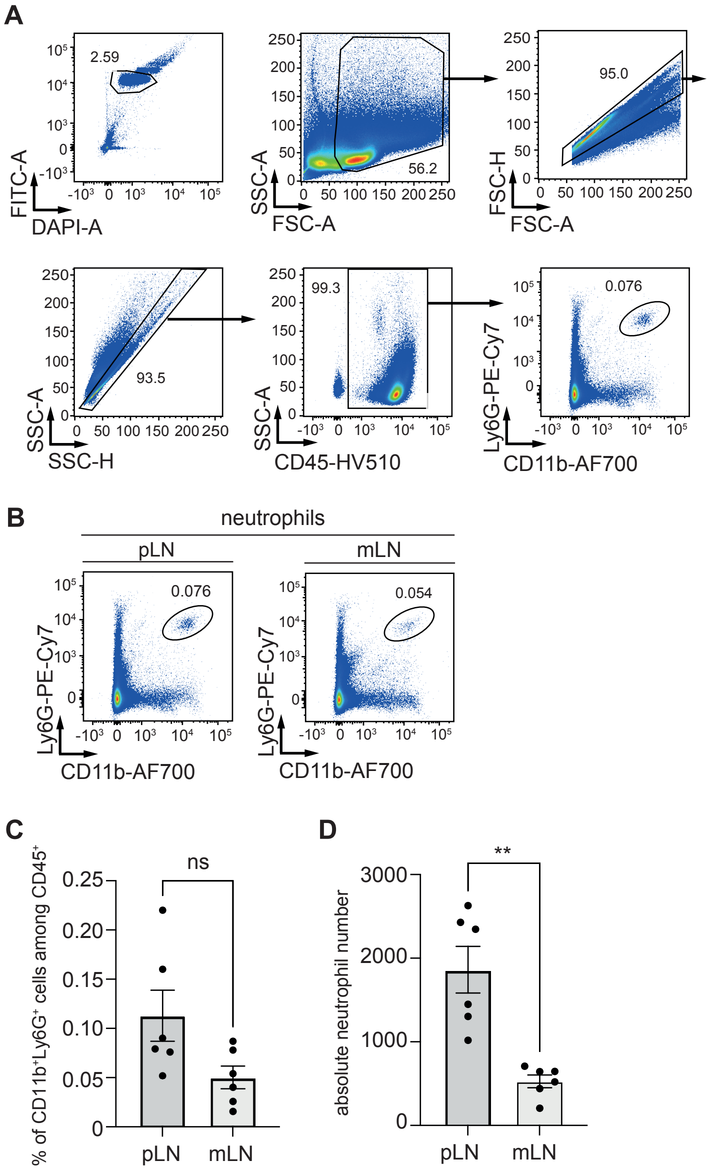

Supplementary Figure 9. Comparison of neutrophil frequency and cell number in pLNs and mLNs. *Related to Figure 4.* Single-cell suspensions were generated from pLNs or mLNs of 7-11 weeks old WT mice, and flow cytometric analysis was carried out. Cells were pre-gated as $CD45^+$ cells. **(A)** Neutrophil gating strategy for lymphoid tissues. The first gate is set on counting beads. **(B)** Representative dot plots depicting the frequency of $CD11b^+Ly6G^+$ cells among $CD45^+$ cells within pLNs and mLNs. **(C)** Bar plot depicting the frequency of $CD11b^+Ly6G^+$ cells among $CD45^+$ cells in pLN and mLNs ($p = 0.0649$). **(D)** Bar plot depicting the absolute cell count for $CD11b^+Ly6G^+$ cells in pLNs and mLNs ($p = 0.0022$). Data pooled from two independent experiments with bars indicating the mean ± SEM ($n = 6$). For statistical analysis, a two-tailed Mann-Whitney U test was used. ns, not significant; mLN, mesenteric lymph node; pLN, skin-draining lymph node. Source data are provided in a Source Data file.

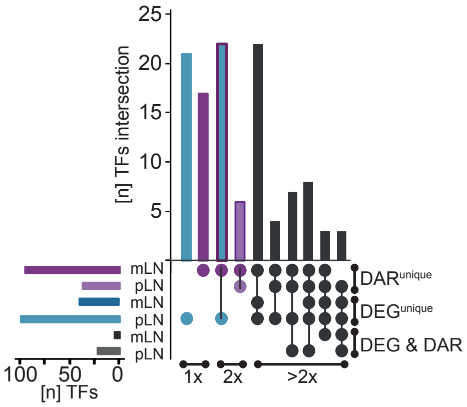

Supplementary Figure 10. Overlap of TFs identified from the epigenomic landscape and transcribed genes. *Related to Figure 4.* $CD45^{-}CD24^{-}CD31^{-}Pdpn^{+}$ SCs were isolated from mLNs and pLNs of 7-12 weeks old SPF mice. Subsequently, RNA-seq or ATAC-seq analyses were performed. DEGs and DARs were identified in mLN vs. pLN pairwise comparisons. Matrix layout for all intersections of putative TFs identified (bar graph left). Circles in the matrix indicate sets that are part of the intersection and the size of the intersection (all intersections with >= 2 are depicted) is indicated in the top bar graph. For both ATAC-seq and RNA-seq, three replicate samples per condition were generated. ATAC-seq, assay for transposase accessible chromatin sequencing; DAR, differentially accessible region; DEG, differentially expressed gene; mLN, mesenteric lymph node; pLN, skin-draining lymph node; SPF, specific pathogen-free; SC, stromal cell; TF, transcription factor.

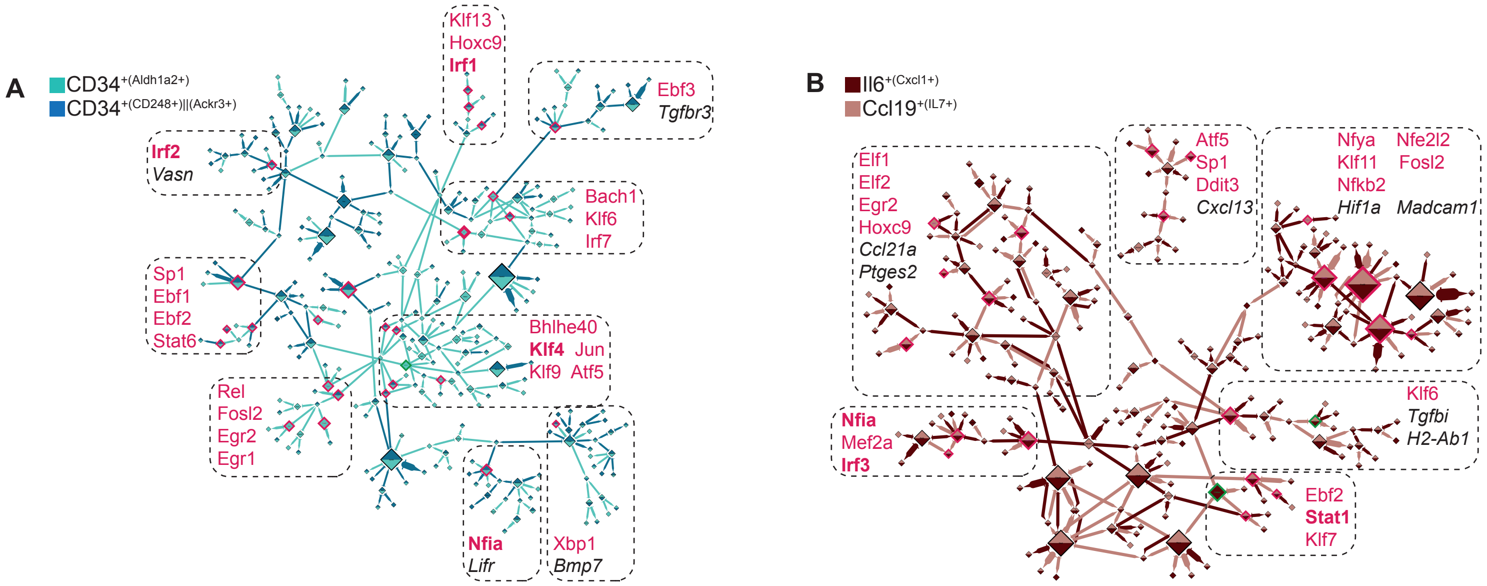

Supplementary Figure 11. TF network analysis revealing putative TFs governing FRC and CD34$^+$ SC biology. *Related to Figure 5.* **(A-B)** Gene regulatory networks based on genes that are dynamically co-expressed along the developmental trajectory, ordering cells within each "branch" per age (see Figure 2). The size of the node is proportional to the node outdegree. TFs inferred from their respective TFBS in accessible chromatin regions (see Figure 2) annotated to the respective node cohort in „pink" | DARs and "black" | SC-associated genes. The color of the edges signifies the terminal branch from which the edge was derived. **(A)** depicts CD34$^+$ SCs. **(B)** depicts FRCs. DAR, differentially accessible region; FRC, fibroblastic reticular stromal cell; SC, stromal cell; TF, transcription factor; TFBS, transcription factor binding sites.

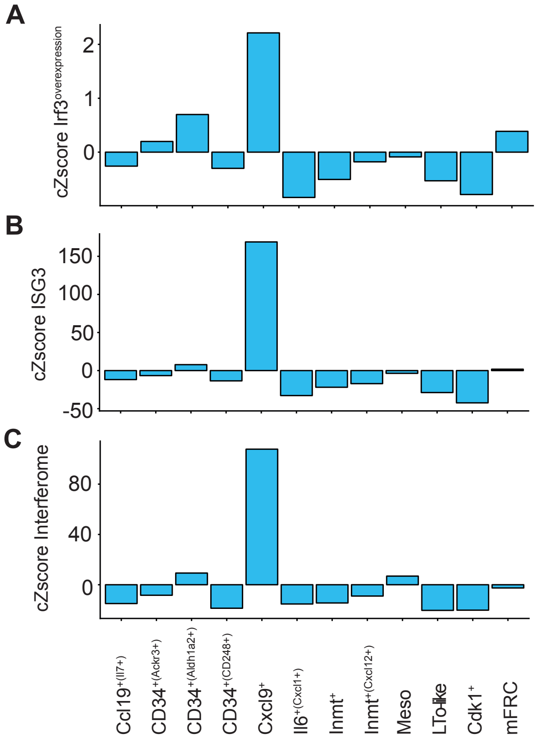

Supplementary Figure 12. Overlap of the *Irf3* TF overexpression signature and IFN response signatures in $Cxcl9^+$ FRCs. *Related to Figure 6.* $CD45^-CD24^-$ cells were isolated from mLNs of day 0, 10, 24, 56 and 300 old SPF-housed mice and subjected to scRNA-seq. Non-endothelial SCs were identified as non-LECs, non-BECs and non-PvCs. **(A)** Bar graph depicting the cZscore overlap of the DEGs identified from *Irf3* overexpression in the C3H10T1/2 cell line with mLN ontogeny cluster signatures (see Figure 1). **(B)** Bar graph depicting the cZscore overlap of the ISG3 signature (PMID 26824662) induced upon viral infection in leucocytes with mLN ontogeny cluster signatures (see Figure 1). **(C)** Bar graph depicting the cZscore overlap of an ISG signature derived from the Interferome database (PMID 23203888) induced upon IFNβ stimulation in fibroblasts with mLN ontogeny cluster signatures (see Figure 1). BEC, blood endothelial cell; cZscore, cumulative Z-score; DEG, differentially expressed gene; FRC, fibroblastic reticular stromal cell; IFN, interferon; LEC, lymphatic endothelial cell; mLN, mesenteric lymph node; PvC, perivascular cell; TF, transcription factor.

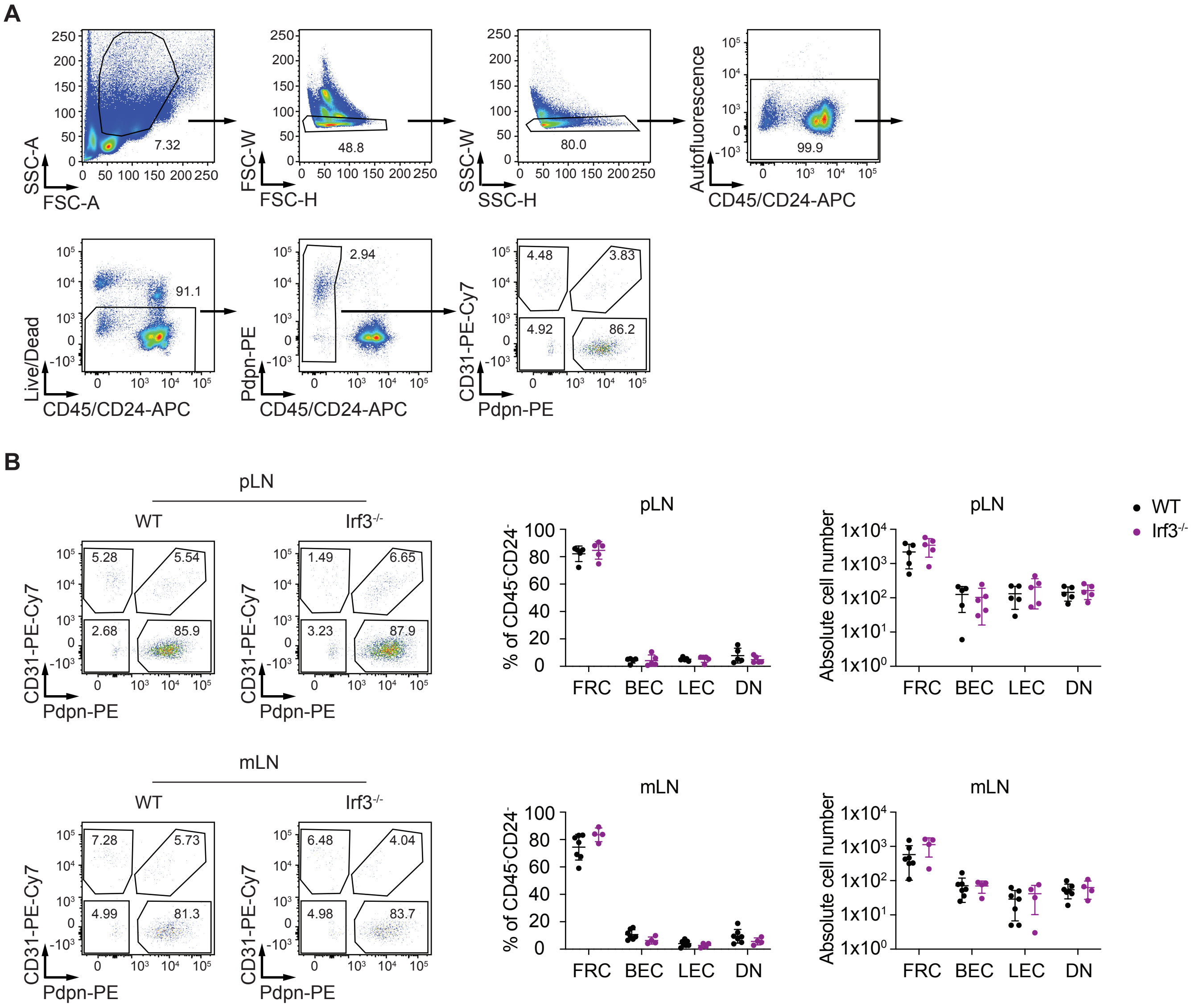

Supplementary Figure 13. Phenotype of SCs in pLNs and mLNs of Irf3$^{-/-}$ mice. *Related to Figure 7.* Single-cell suspensions were generated from pLNs or mLNs of 12-14 weeks old WT and Irf3$^{-/-}$ male mice and flow cytometric analysis was carried out. **(A)** SC gating strategy. **(B)** Representative pseudocolor plots depicting frequencies of FRCs (Pdpn$^+$CD31$^-$), BECs (Pdpn$^-$CD31$^+$), LECs (Pdpn$^+$CD31$^+$) and DNs (Pdpn$^-$CD31$^-$) among CD45$^-$CD24$^-$ cells within pLNs and mLNs (left). Scatter dot plots summarize frequencies (middle) and absolute cell numbers (right) of indicated subsets in pLNs and mLNs. Data pooled from two independent experiments with bars indicating mean ± SD (n = 5, pLN WT; n=5, pLN Irf3$^{-/-}$; n=7, mLN WT; n=4, mLN Irf3$^{-/-}$). For statistical analysis, a two-tailed Mann-Whitney test was used for the direct comparison between WT and Irf3$^{-/-}$. BECs, blood endothelial cells; DNs, double negative cells; FRCs, fibroblastic reticular stromal cells; LECs, lymphatic endothelial cells; mLN, mesenteric lymph node; pLN, skin-draining lymph node; SCs, stromal cells; WT, wild type. Source data are provided in a Source Data file.
