# Supplementary material for: Postnatal expansion of mesenteric lymph node stromal cells towards reticular and CD34+ stromal cell subsets
Source: Nat Commun. 2022 Nov 24;13:7227. doi: 10.1038/s41467-022-34868-4 (PMC9700677; doi:10.1038/s41467-022-34868-4)
Supplement: Supplementary file 2 — Description of Additional Supplementary Files [file 41467_2022_34868_MOESM2_ESM.pdf]

**Description of Additional Supplementary Files**

File Name: Supplementary Data 1
Description: Transcriptional signature genes per stromal cell subset, CD45-CD24- cells were isolated from mLNs of day 0, 10, 24, 56 and 300 old SPF-housed mice and subjected to scRNA-seq. SCs were identified as non-LECs, non-BECs. Table summarizes marker DEGs up-regulated for each cluster, pct.1, percent of cells expressing gene in respective cluster; pct.2, percent of cells expressing gene in respective reference (1=100%)

File Name: Supplementary Data 2
Description: Transcriptional signature genes per stromal cell subset, CD45-CD24- cells were isolated from mLNs of day 0, 10, 24, 56 and 300 old SPF-housed mice and subjected to scRNA-seq, non-endothelial SCs were identified as non-LECs, non-BECs, non-PvC and non-adjacent tissue cells (adja). Table summarizes marker DEGs up-regulated for each cluster, pct.1, percent of cells expressing gene in respective cluster; pct.2, percent of cells expressing gene in respective reference (1=100%)

File Name: Supplementary Data 3
Description: Commensal-dependent differentially methylated regions. CD45-CD24-CD31-Pdpn+ SCs were isolated from mLNs and pLNs of GF or SPF mice and WGBS analysis were performed.

File Name: Supplementary Data 4
Description: Accessible chromatin loci in non-endothelial SC. CD45-CD24-CD31-Pdpn+ SCs were isolated from mLNs and pLNs of GF or SPF mice. ATAC-seq analysis was performed. DARs were identified in colonization- (SPF vs. GF) and location-dependent (mLN vs. pLN) pairwise comparisons.

File Name: Supplementary Data 5
Description: Accessible chromatin loci in non-endothelial SC. CD45-CD24-CD31-Pdpn+ SCs were isolated from mLNs and pLNs of GF or SPF mice ATAC-seq analysis was performed. DARs were identified in colonization- (SPF vs. GF) and location-dependent (mLN vs. pLN) pairwise comparisons.
