# Supplementary material for: Postnatal expansion of mesenteric lymph node stromal cells towards reticular and CD34+ stromal cell subsets
Source: Nat Commun. 2022 Nov 24;13:7227. doi: 10.1038/s41467-022-34868-4 (PMC9700677; doi:10.1038/s41467-022-34868-4)
Supplement: Supplementary file 8 — Reporting Summary [file 41467_2022_34868_MOESM8_ESM.pdf]

nature research

Corresponding author(s): Prof. Jochen Huehn

Last updated by author(s): Oct 27, 2022

Reporting Summary

Nature Research wishes to improve the reproducibility of the work that we publish. This form provides structure for consistency and transparency in reporting. For further information on Nature Research policies, see our Editorial Policies and the Editorial Policy Checklist.

Statistics

For all statistical analyses, confirm that the following items are present in the figure legend, table legend, main text, or Methods section.

n/a	Confirmed	
☐	☒	The exact sample size (n) for each experimental group/condition, given as a discrete number and unit of measurement
☐	☒	A statement on whether measurements were taken from distinct samples or whether the same sample was measured repeatedly
☐	☒	The statistical test(s) used AND whether they are one- or two-sided<br>*Only common tests should be described solely by name; describe more complex techniques in the Methods section.*
☒	☐	A description of all covariates tested
☐	☒	A description of any assumptions or corrections, such as tests of normality and adjustment for multiple comparisons
☐	☒	A full description of the statistical parameters including central tendency (e.g. means) or other basic estimates (e.g. regression coefficient) AND variation (e.g. standard deviation) or associated estimates of uncertainty (e.g. confidence intervals)
☐	☒	For null hypothesis testing, the test statistic (e.g. F, t, r) with confidence intervals, effect sizes, degrees of freedom and P value noted<br>*Give P values as exact values whenever suitable.*
☒	☐	For Bayesian analysis, information on the choice of priors and Markov chain Monte Carlo settings
☐	☒	For hierarchical and complex designs, identification of the appropriate level for tests and full reporting of outcomes
☐	☒	Estimates of effect sizes (e.g. Cohen's d, Pearson's r), indicating how they were calculated

Our web collection on statistics for biologists contains articles on many of the points above.

Software and code

Policy information about availability of computer code

Data collection	VS-ASW-FL (version 2.9 and 2.9.2).
Data analysis	VS-ASW-FL (version 2.9 and 2.9.2)<br>FlowJo (version 10.8.1)<br>FastQC (version 0.11.1)<br>Trim_galore (version 0.6.5)<br>Cutadapt (version 1.4)<br>BSMAP (version 2.4.3)<br>Bsseq (version 1.16)<br>STAR (version 2.5.3a and 2.7.3a)<br>DESeq2 (version 1.22)<br>macs2 (version 2.2.6)<br>ChIPpeakANNO (version 3.6.5)<br>Homer (version 4.9)<br>Tophat2 (version 1.2.0)<br>HTSeq (version 0.12.4)<br>Cell Ranger (version 2.0.0 and 6.0.0)<br>Seurat (version 2.3.3 and 4.0.1)<br>Monocle (version 2.18.0)<br>igraph (version 1.2.2)<br>TopGO (version 2.12)<br>BRB-seqTools (version 1.4)<br>R (version 3.4.1 and 4.0.5)

GraphPad Prism (version 8.0.1)

The code utilized for this study is available at https://github.com/jpezoldt/LymphNode_StromaCell_Ontogeny

For manuscripts utilizing custom algorithms or software that are central to the research but not yet described in published literature, software must be made available to editors and reviewers. We strongly encourage code deposition in a community repository (e.g. GitHub). See the Nature Research guidelines for submitting code & software for further information.

Data

Policy information about availability of data

All manuscripts must include a data availability statement. This statement should provide the following information, where applicable:

- Accession codes, unique identifiers, or web links for publicly available datasets
- A list of figures that have associated raw data
- A description of any restrictions on data availability

ATAC-seq, WGBS, RNA-seq, scRNA-seq and BRB-seq raw and processed data generated in this study have been deposited in the NCBI GEO database under accession code GSE172526 [https://www.ncbi.nlm.nih.gov/geo/query/acc.cgi?acc=GSE172526].

Field-specific reporting

Please select the one below that is the best fit for your research. If you are not sure, read the appropriate sections before making your selection.

☒ Life sciences ☐ Behavioural & social sciences ☐ Ecological, evolutionary & environmental sciences

For a reference copy of the document with all sections, see nature.com/documents/nr-reporting-summary-flat.pdf

Life sciences study design

All studies must disclose on these points even when the disclosure is negative.

Sample size	Sample size was chosen based on previous experience and according to standards of the field.
Data exclusions	For WGBS, one mLN-GF and one pLN-GF replicate outlier was excluded from the study.
Replication	For all FACS analyses, at least two replicates were carried out in independent experiments.<br>For bulk sequencing approaches, at least two replicates were carried out in independent experiments. All attempts were successful except the datasets for WGBS mentioned in section “Data exclusions”.
Randomization	Randomization was not required for this study.
Blinding	Investigator blinding was not required for this study.

Reporting for specific materials, systems and methods

We require information from authors about some types of materials, experimental systems and methods used in many studies. Here, indicate whether each material, system or method listed is relevant to your study. If you are not sure if a list item applies to your research, read the appropriate section before selecting a response.

Materials & experimental systems

n/a	Involved in the study	
☐	☒	Antibodies
☐	☒	Eukaryotic cell lines
☒	☐	Palaeontology and archaeology
☐	☒	Animals and other organisms
☒	☐	Human research participants
☒	☐	Clinical data
☒	☐	Dual use research of concern

Methods

n/a	Involved in the study	
☒	☐	ChIP-seq
☐	☒	Flow cytometry
☒	☐	MRI-based neuroimaging

Antibodies

Antibodies used	Fluorochrom-conjugated anti-CD11b (clone M1/70, AF700, eBioscience Cat. #56 0112 82, 1:800), anti CD24 (clone M1/69, APC, BioLegend Cat. #101814, 1:800), anti CD31 (clone 390, PE Cy7, BioLegend Cat. #102418, 1:1000), anti CD45 (clone 30 F11, APC, BioLegend Cat. #103112, 1:400), anti CD45 (clone 30 F11, HV510, BD Cat. #561487, 1:400), anti gp38 (clone 8.1.1, PE, BioLegend Cat. #127408, 1:1000), anti-Ly6G (clone 1A8, PE-Cy7, BD Cat. #560601, 1:1000), and Ter119 (clone Ly-76, APC, Biolegend Cat. #116212,

	1:500) were utilized in this study. For FcγR blocking, anti-CD16/CD32 (clone 2.4G2, BioXcell, Cat. #BE0008, 1:100) was used.
Validation	Before use, all antibodies were validated by titration using primary cells expressing the respective target antigens.

Eukaryotic cell lines

Policy information about cell lines

Cell line source(s)	HEK 293T cells (ATCC Cat. No. SD-3515); C3H10T1/2 cells (ATCC Cat. No. CCL 226)
Authentication	Morphological assessment via microscopy and expression profiling were used to ensure origin of cell line.
Mycoplasma contamination	Cell lines were tested negative for mycoplasma contamination before the experiments were performed.
Commonly misidentified lines (See ICLAC register)	Commonly misidentified cell lines were not used in the study.

Animals and other organisms

Policy information about studies involving animals; ARRIVE guidelines recommended for reporting animal research

Laboratory animals	CD90.1 (BALB/c), Foxp3hCD2 (C57BL/6), Irf3-/- (C57BL/6) and C57BL/6 mice of both genders and an age between 0 and 300 days were used in the study.
Wild animals	No wild animals were used in the study.
Field-collected samples	No field collected samples were used in the study.
Ethics oversight	All mice were housed and handled in accordance with good animal practice as defined by FELASA and the national animal welfare body GV-SOLAS.

Note that full information on the approval of the study protocol must also be provided in the manuscript.

Flow Cytometry

Plots

Confirm that:

☒ The axis labels state the marker and fluorochrome used (e.g. CD4-FITC).

☒ The axis scales are clearly visible. Include numbers along axes only for bottom left plot of group (a 'group' is an analysis of identical markers).

☒ All plots are contour plots with outliers or pseudocolor plots.

☒ A numerical value for number of cells or percentage (with statistics) is provided.

Methodology

Sample preparation	For neutrophil isolation, skin-draining pLNs (inguinal and axilliary) or mLNs (small intestinal and colon/caecum draining) were isolated. Resected LNs were gently meshed through a 30 µm strainer to create single-cell suspensions.<br>For stromal cell isolation, skin-draining pLNs (inguinal and axillary) or mLNs (small intestinal and colon/caecum-draining) were resected and enzymatically digested. After digestion, CD45- cells were enriched by autoMACS separation.
Instrument	FACSymphony A5, LSR Fortessa
Software	FlowJo (BD)<br>GraphPad Prism (GraphPad)
Cell population abundance	Neutrophil abundance among CD45+ cells was around 0.02-0.25%.<br>Stromal cell abundance among CD45- enriched cells was around 3%.
Gating strategy	Neutrophil gating: FSC-SSC, FSC-FCSH, SSC-SSCH, SSC-CD45, then neutrophils were identified as Ly6C+CD11b+ cells.<br>Stromal cell gating: FSCA-SSCA, FSCH-FCSW, SSCH-SSCW, autofluorescence, live/dead, CD45-CD24-, then stromal cell subsets were gated according to Pdpn and CD31 expression.

☒ Tick this box to confirm that a figure exemplifying the gating strategy is provided in the Supplementary Information.
